# Complex impacts of gallstone disease on metabolic syndrome and nonalcoholic fatty liver disease

**DOI:** 10.3389/fendo.2022.1032557

**Published:** 2022-11-23

**Authors:** Jingting Lyu, Qinghong Lin, Zhongbiao Fang, Zeling Xu, Zhengtao Liu

**Affiliations:** ^1^ Shulan International Medical College, Zhejiang Shuren University, Hangzhou, Zhejiang, China; ^2^ NHC Key Laboratory of Combined Multi-Organ Transplantation, Key Laboratory of the Diagnosis and Treatment of Organ Transplantation, CAMS, First Affiliated Hospital, School of Medicine, Zhejiang University, Hangzhou, Zhejiang, China; ^3^ Key Laboratory of Organ Transplantation, Zhejiang Province, First Affiliated Hospital, School of Medicine, Zhejiang University, Hangzhou, Zhejiang, China; ^4^ Shulan (Hangzhou) Hospital, Hangzhou, China

**Keywords:** gallstone disease, metabolic syndrome, nonalcoholic fatty liver disease, cholecystectomy, insulin resistance, meta-analysis

## Abstract

**Background:**

Patients with gallstone disease (GSD) often have highly co-occurrence with metabolic syndrome (MetS) and Nonalcoholic fatty liver disease (NAFLD) both associated with insulin resistance (IR). Meanwhile, highly prevalence of NAFLD was found in patients who received cholecystectomy. However, the associations of GSD with MetS, NAFLD is inconsistent in the published literature. And risk of cholecystectomy on NAFLD is unclear.

**Methods:**

We searched the Medline EMBASE and WOS databases for literature that met our study topic. To be specific, studies with focus on associations between GSD and MetS/NAFLD, and risk evaluation on cholecystectomy and NAFLD incidence were enrolled for further analysis. The random effect model was used to calculate the combined relative ratio (RR) and odds ratio (OR)and 95% confidence interval (CI).

**Results:**

Seven and six papers with focus on connections between GSD and NAFLD/MetS prevalence. Correspondingly, seven papers with focus on risk of cholecystectomy on NAFLD occurrence were also enrolled into meta-analysis. After pooling the results from individual study, patients with GSD had higher risk of MetS (OR:1.45, 95%CI: 1.23-1.67, I^2^ = 41.1%, P=0.165). Risk of GSD was increased by 52% in NAFLD patients (pooled OR:1.52, 95%CI:1.24-1.80). And about 32% of increment on NAFLD prevalence was observed in patients with GSD (pooled OR: 1.32, 95%CI:1.14-1.50). With regard to individual MetS components, patients with higher systolic blood pressure were more prone to develop GSD, with combined SMD of 0.29 (96%CI: 0.24-0.34, P<0.05). Dose-response analysis found the GSD incidence was significantly associated with increased body mass index (BMI) (pooled OR: 1.02, 95%CI:1.01-1.03) in linear trends. Patients who received cholecystectomy had a higher risk of post-operative NAFLD (OR:2.14, 95%CI: 1.43-2.85), P<0.05). And this impact was amplified in obese patients (OR: 2.51, 95%CI: 1.95-3.06, P<0.05).

**Conclusion:**

Our results confirmed that controls on weight and blood pressure might be candidate therapeutic strategy for GSD prevention. And concerns should be raised on *de-novo* NAFLD after cholecystectomy.

## 1 Introduction

Gallstone disease (GSD) is a significant burden in health care around the world (1). GSD is the second largest digestive disease after gastroesophageal reflux disease in the United States (2). GSD caused great pain to adults (3). Although the incidence was much higher than that of children, it tended to be younger (4). Its incidence is also high in the worldwide population with a prevalence of 5-25% in Westerners (5) and 3-15% in Asians (6). In spite of lower mortality, much payment should be listed from medical insurance for hospitalization and treatment for GSD patients (7). Cholecystectomy is the most common surgical procedure for the treatment of cholelithiasis and its complications in the world, where laparoscopic surgery was used in about 90% of cases (5). Risk factors for GSD such as cholecystitis (acute/chronic), symptomatic cholelithiasis, biliary dyskinesia, acalculous cholecystitis, gallstone pancreatitis and gallbladder masses/polyps can be treated by cholecystectomy (8). In addition to common bile duct injury, bile leakage (9), bleeding, indigestion and vague non-colic abdominal pain (10), cholecystectomy can further cause a series of metabolic changes such as increased serum triglyceride, rising very-low-density-lipoprotein levels (11, 12) and metabolic syndrome in cardiovascular diseases like type 2 diabetes and hypertension.

Clinically, insulin resistance (IR) is defined as the inability of insulin to keep blood glucose levels in a healthy range (13). However, apart from regulating glucose metabolism, insulin was also involved in other metabolic activities in the body (14). IR played a crucial role in metabolic disorders such as metabolic syndrome (MetS) and hepatic steatosis (14, 15). MetS and GSD have common risk factors, and the greatest correlation is abdominal obesity and insulin resistance (16). Nonalcoholic fatty liver disease (NAFLD) represents an excessive accumulation of adipocytes in the liver as presentation of IR in liver. It often coexisted with GSD (17). Current research showed that insulin resistance and GSD can influence each other (18). That is, IR promoted GSD, and GSD in turn aggravated IR (18). Results from large cohort of non-diabetic Korean men found systemic IR as independent predictor for GSD (19). The most important way insulin resistance affected GSD was to disrupt the metabolism of cholesterol in the body (20). A study shown that both MetS and NAFLD can accelerate the increase of cholesterol synthesis in the body, and the excessive secretion of bile cholesterol was related to the increase of bile lithogenicity (20).

Conversely, systemic glucose and lipid metabolism can be regulated by gallbladder (21). The gallbladder helps to maintain glucose, lipids and homeostasis (21). When GSD occurred, cholesterol in bile was increased with lowered phospholipids and bile acid (21). Both cholecystectomy and GSD had adverse effects on insulin sensitivity (22). Moreover, there were persistent defects in the regulation of liver lipid metabolism in patients undergoing cholecystectomy (22). Therefore, cholecystectomy particularly influenced the occurrence and development of NAFLD.

In view of the tight relationship between GSD and metabolic derangements, many studies were performed with topics on associations between GSD and MetS/NAFLD occurrence (23–36). Otherwise, the impacts of cholecystectomy on post-operative NAFLD were also assessed in previous studies (12, 31, 37–41). However, the above relationships were still controversial with difference across individual studies. Several EBM papers were published to illustrate the associations between GSD and metabolic derangements (42, 43). After careful evaluation, we found several defects for these reviews. To be specific, in literature by Veeravich (42) and Jiang (43) etc, authors only calculated the quantitative correlations between GSD and NAFLD/MetS without considerations on direction of these two covariates, which can’t avoid potential bias inevitably. Otherwise, Jiang et al. (42) only referred patients with higher BMI had higher susceptibility to develop GSD. But in-depth dose-response analysis was not performed in prior EBM study to illustrate the continuous effects of BMI variations on GSD incidence. Hence, to timely update assessment, literature involved on GSD and NAFLD/MetS need to be categorized by directions to analyze the bidirectional relationship between GSD and NAFLD/MetS. And in-depth dose-response analysis should be performed to show the continuous risk of quantitative metabolic variables on GSD risk.We conducted systematic review and meta-analysis based on the existing literature for more effective evidence on prevention of GSD and post-cholecystectomyic metabolic complications.

## 2 Materials and methods

### 2.1 Search strategy

A Meta-analysis was conducted according to the guidelines of the Preferred Reporting Items for Systematic Reviews and Meta-analyses (PRISMA) (see checklist S1, flow diagram S1 and abstract checklist S1) (44). A relevant literature search was conducted using Medline, Embase, and Web of Science (WOS) databases from the date of inception to 24 July 2022 (without language restrictions). The following terms were used to search literatures: “gallstones disease”; “cholelithiasis”; “Metabolic syndrome”; “syndrome X”; “insulin resistance syndrome;” “MetS”; “Nonalcoholic fatty liver disease;” “nonalcoholic Steatohepatitis”; “NAFLD”; “NASH”; “cholecystectomy”; and “Laparoscopic cholecystectomy.” If relevant literatures were omitted, additional manual retrieval was performed. The search strategy for the database is available in **Table S1**.

### 2.2 Study selection and data extraction

Eligibility criteria: 1) Published retrospective, prospective cohort studies and cross-sectional studies. 2) GSD / METS / NAFLD was the testing group's endpoint. 3) MetS diagnostic criteria for the study were given and the diagnosis of cholelithiasis needed to be confirmed by imaging or surgery. 4) The odds ratio (OR) / relative ratio (RR) / Hazard Ratio (HR) and corresponding 95% confidence intervals (CI) may be derived from studies or can be calculated. Studies were excluded if 1) literature was not the above research type, or the unpublished; 2) The subjects were not related to GSD/METS/NAFLD (no interest of subjects); 3) The disease outcomes were not just GSD/METS/NAFLD but with organic lesions, liver dysfunction or viral hepatitis in the hepatobiliary system (no interest of outcomes).

The study characteristics were extracted from all the literatures: first author; publication year; country and ethnicity of data origin; study design type; enrolled study population (including number of cases and total number); disease outcome; definition of MetS and NAFLD; risk of disease; the mean ± SD of MetS components such as body mass index (BMI), blood pressure (systolic pressure, diastolic pressure), waist circumference (WC), triglyceride (TG), fasting blood glucose (FBG), high density lipoprotein cholesterol (HDL-C) in case and control group; the calculation method of origin data; and adjusted covariates (the risks with the most extensive covariate adjusted were included to avoid potential bias).

### 2.3 Quality assessment

A preliminary assessment of the quality of each study was evaluated by two authors (JTL and QHL), respectively using the Newcastle-Ottawa Scale (NOS) (45). NOS consists of three main components, including participant selection, interstudy comparability and outcome assessment, corresponding to four, three and two stars (**Table S2**). Nine stars represent the highest quality paper, and a score of six stars is considered high-quality research. Meanwhile, we also carried out GRADE evaluation to assess our results quality (**Figures S2**, **S3**). When there are differences between the two authors, the original paper is re-evaluated by a third author (ZLX).

### 2.4 Statistical analysis

We initially combined the results of the included studies with a random effect model (Inverse variance), and chose OR (for cross-sectional study) and RR (for prospective cohort study))and 95% confidence intervals to quantify the relationship between Gallstone and MetS, gallstone and NAFLD, and cholecystectomy and NAFLD. If heterogeneity is significant, the random-effects model will be used, and heterogeneity will be assessed by I^2^ statistics (low, high and medium heterogeneity of I^2^ are defined as 25%, 50% and 75%, respectively (46).

To explore the relationship between GSD and MetS/NAFLD, we first compared the combined OR of end-stage (MetS/NAFLD) with or without GSD and the combined OR of end-stage (GSD) with or without GSD. Secondly, according to the different BMI intervals provided in the two reports (29, 30), the midpoints of each interval’s upper and lower bounds were set as approximate median or mean. When the highest category was available, 1.2 times the category’s low value was allocated (47). Using generalized least squares (GLST) calculations, BMI levels were estimated for each 1kg/m^2^ increase, and then OR increments from both studies were combined to reflect the linear dose-response risk for GSD incidence. On the basis of information from reviewed studies, pooled standardized mean differences (SMD) of specific individual MetS components connected to the GSD occurrence were analyzed. In addition, a subgroup analysis was performed to determine the impact of potential confounding factors. Sensitivity analysis was carried out to look into the effects that a single study could have had on the outcomes.

When discussing the relationship between cholecystectomy and NAFLD, the combined RR and RR of NAFLD patients with or without cholecystectomy were also compared. Subsequently, subgroup analysis and meta-regression were performed to explore the heterogeneity of potential sources further.

To calculate any potential publication bias, Egger’s test was employed. P<0.05 was deemed significance. All of the statistical analyses were performed using Stata 15.0 version software (Stata Corp, College Station, TX, USA).

Our study flow diagram was shown in **Figure S1**.

## 3 Results

### 3.1 Search results and quality assessment results

**Figure 1:**
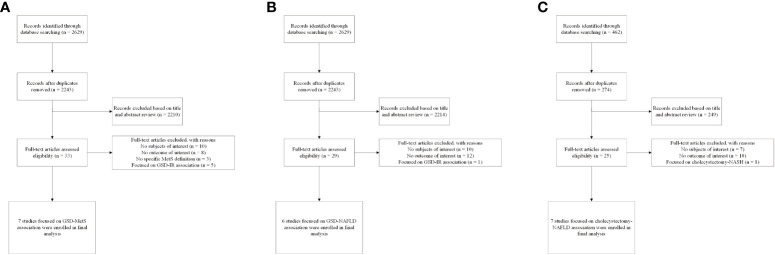
Flow diagram of eligible literature selection **(A)** Literature on association between GSD and MetS; **(B)** Literature on association between GSD and NAFLD; **(C)** Literature on association between cholecystectomy and NAFLD. GSD, gallstone disease; MetS, metabolic syndrome; NAFLD, nonalcoholic fatty liver disease; IR, insulin resistance.

The flow diagram of the meta-analysis registration study was shown in **Figure 1**. After excluding 894 duplicates from three databases (Medline, Embase, and WOS), we screened 3210 potentially relevant articles. The number of eligible articles finally registered was 19 (seven for GSD-MetS, six for GSD-NAFLD and six for cholecystectomy-NAFLD) with a high degree of uniformity among the evaluators (Cohen’s Kappa =0.766). A manual search of references and related reviews of published studies revealed no additional studies. According to the NOS rating system, all included studies were assessed to be of good quality. Participants’ NOS scores ranged from 6 to 9, with an average score of 7.84. The results of NOS quality assessment scores are shown in **Table S3**. The results of GRADE evaluation reported low level of our enrolled articles because they were observational studies (**Figures S2**, **S3**).

### 3.2 Characteristics of the enrolled studies

**Figure 2:**
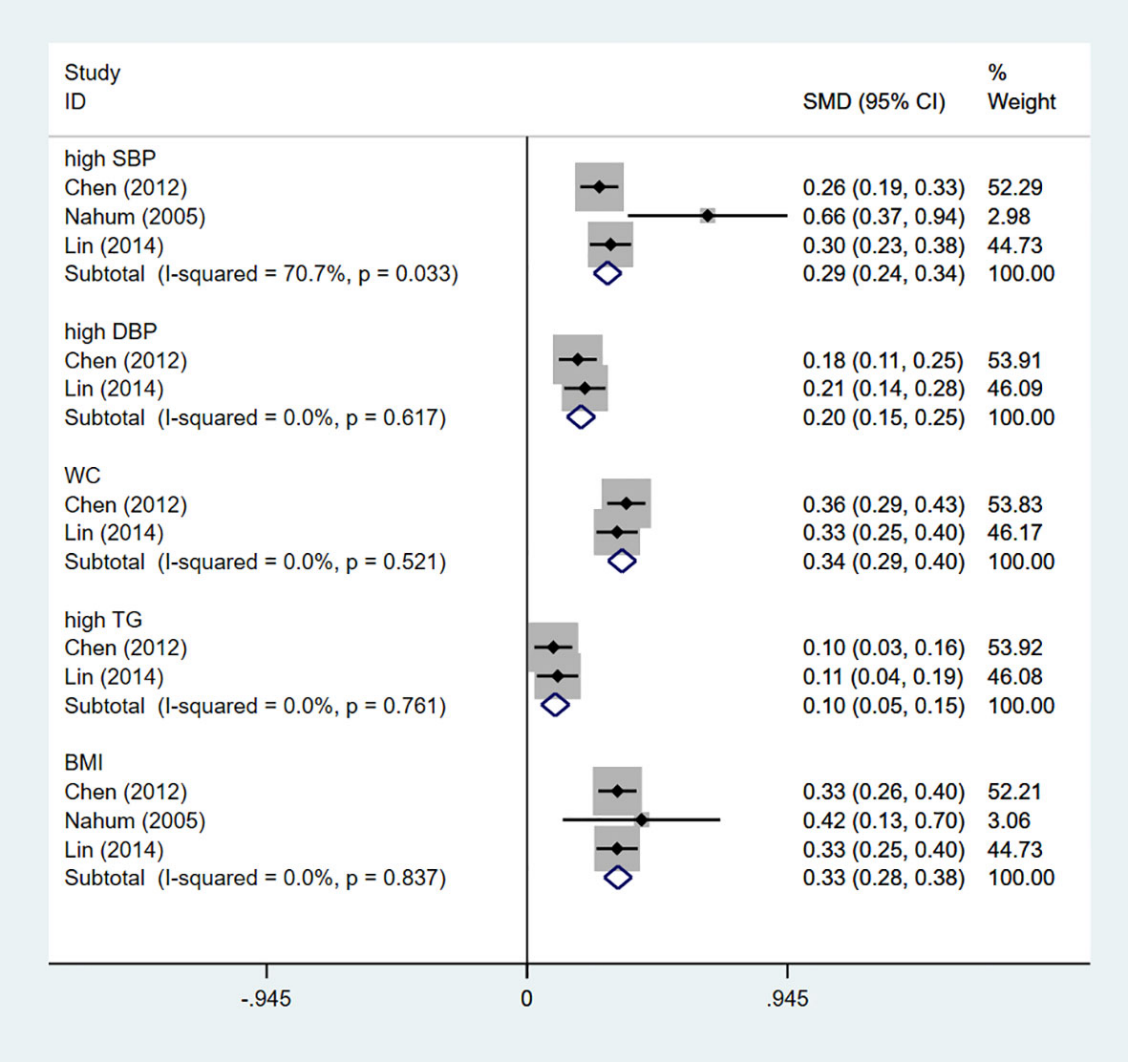
The influence of single MetS component on GSD risk. GSD, gallstone disease; MetS, metabolic syndrome; SBP, systolic blood pressure; DBP, diastolic blood pressure; WC, waist circumference; TG, triglyceride; BMI, body mass index.

The study characteristics are presented in **Table 1**. Seven articles on the relationship between GSD and MetS were included in the meta-analysis, of which two prospective cohort studies reported the effect of MetS on the incidence of GS. Four cross-sectional and one prospective cohort studies reported the effect of GSD on the incidence of MetS. East Asians, Hispanics and Caucasians participated in five, one and one studies. Sample size varies widely between studies, with some fewer than 300 people and some as many as 200,000, for a total of 246,006 participants. Among the participants, 3,802 had GSD, with a prevalence ranging from 1.41% to 4.77%, and 2,034 had MetS, with a prevalence ranging from 6.09% to 35.02%. Three reported whether a single MetS component would affect the incidence of GSD and presented by standardized mean differences (SMD), we merge the results as shown in **Figure 2**. Regarding to the diagnostic criteria of MetS, the definition provided by the National Cholesterol Education Program Adult treatment Group III (NCEPATPIII) (48) in four studies and one used modified NCEP-ATP III. Alternatively, Chinese Diabetes Society (CDS) (49), the International Diabetes Federation (IDF) (50), and Taiwan National Health Department criteria (51) were used for the enrolled studies (**Table S4**). Four, one, and one studies’ results were calculated using multivariate logistic regression, the Chi-Square test, multiple GEE, and Cox proportional hazard models.

**Table 1:** Characteristics of the nineteen studies included in meta-analysis.

First author, publication year [ref]	Country	Ethnicity	Study design	Enrolled study population(case/total)	Outcome	MetS definition	NAFLD diagnose	MetS components	OR/RR/HR	Mean ± SD (case/control)	Calculation method	Adjusted covariates
Chen et al. 2012 (25)	China	East Asian	Cross-sectional	918/7570 without MetS	MetS	NCEP-ATP-III on the Asia			OR: 1.29(1.09-1.52)		Multivariate logistic regression model	Age
								BMI		26.3 ± 3.0 25.2 ± 3.4		
								WC		91.6 ± 9.4 87.8 ± 10.7		
								SBP		123.6 ± 14.3 119.8 ± 14.5		
								DBP		74.1 ± 9.8 72.2 ± 10.4		
								FBG		5.39 ± 1.37 5.11 ± 1.04		
								TG		201.1 ± 183.3 183.7 ± 182.7		
Nahum et al. 2005 (29)	USA	Hispanics	Cross-sectional	65/245 without MetS	MetS	NCEP-ATPIII			OR: 2.79(1.46-5.33)		Multivariate logistic regression model	Age and sex
								WC	OR: 3.61(1.95-6.71)			
								BMI		28.4 ± 5.7 26.3 ± 4.8		
								SBP		15.7 ± 2.2 14.4 ± 1.9		
Lin et al. 2014 (27)	China	East Asian	Cross-sectional	734/12050 without MetS	MetS	Taiwan criteria			OR: 1.61(1.336-1.898)		Multivariate logistic regression model	Age and sex
								WC		84.4 ± 10.1 81.2 ± 9.9		
								SBP		127.8 ± 17.8 122.6 ± 17.1		
								DBP		81.0 ± 11.1 78.8 ± 10.6		
								HDL-C		46.1 ± 12.1 49.2 ± 12.8		
								TG		126.8 ± 111.9 115.1 ± 10.5		
Naim et al. 2011 (28)	Turkey	Caucasian	Cross-sectional	217/217 without MetS	MetS	NCEP-ATPIII			OR: 1.434(1.222-1.846)		Multivariate logistic regression model	Age, MetS, DM, large WC, HOMA-IR, gallstone size and BMI
Amit et al. 2019 (24)	India	East Asian	Prospective cohort	100/200 without MetS	MetS	NCEP-ATPIII			RR: 1.313(1.107-1.556)		Chi- Square test model	
Kim et al. 2021 (23)	Korea	East Asian	Prospective cohort	2929/ 207850 without GSD	GSD	IDF			HR: 1.39(1.05-1.85)		Cox proportional hazard model	Age, sex, eGFR, GGT, smoking, alcohol intake and physical activity
Zhu et al. 2016 (26)	China	East Asian	Prospective cohort	873/18291 without GSD	GSD	CDS			RR: 1.25(1.06-1.49)		Multiple GEE model	Age
Kim et al. 2009 (30)	Korea	East Asian	Cross-sectional	6085/ 34574 without MetS	MetS	NCEP-ATPIII		BP	OR: 1.67(1.58-2.00)		Multivariate logistic regression model	Age, MetS, DM, large WC, HOMA-IR, gallstone size and BMI
Koller et al 2012 (32)	Slovakia	Northern European	Cross-sectional	198/482 without GSD	GSD		USG		OR: 1.78(1.16-2.73)		Multivariate logistic regression model	Age, gender, BMI, smoking, alcohol drinking, regular exercise,
												and DM, hypertension, eGFR and HDL-C
Koller et al 2012 (9)	Slovakia	Northern European	Cross-sectional	166/482 without NAFLD	NAFLD		USG		OR: 1.92(1.24-2.96)		Multivariate logistic regression model	Age, gender, BMI, smoking, alcohol drinking, regular exercise,
												and DM, hypertension, eGFR, total cholesterol, triglyceride, and HDL-C
Chang et al 2018 (31)	Korea	East Asian	Prospective cohort	214446/ 283446 without GSD	GSD		USG		HR: 1.26(1.17-1.35)		Multivariate logistic regression model	BMI, smoking, alcohol intake, exercise, total calorie intake, hypertension,
												and diabetes, dyslipidemia, LDL-C, HDL-C, triglycerides and HOMA-IR
				4073/ 218519 without NAFLD	NAFLD		USG		HR: 1.14(1.07-1.22)			
Young et al. 2019 (36)	Korea	East Asian	Cross-sectional	355/7886 without GSD	GSD		USG		OR: 1.48(0.875-1.485)		Binary logistic regression model	Age, sex, grade of fatty liver disease, BMI, fasting blood glucose, and total cholesterol, LDLs, HDLs, triglycerides
Liu et al. 2014 (34)	China	East Asian	Prospective cohort	498/11200 without GSD	GSD		USG		RR: 1.33(1.00-1.53)		Multiple GEE model	Age, BMI, SBP, ALB, GLO, TG and GLU
Qiao et al. 2017 (35)	China	East Asian	Cross-sectional	919/7583 without NAFLD	NAFLD		USG		OR: 1.28(1.07-1.52)		Multivariate logistic regression model	BMI≥24, hyper- lipidaemia, and hypertension
Lee et al. 2014 (33)	China	East Asian	Cross-sectional	768/12033 without NAFLD	NAFLD		USG		OR: 1.32(1.04-1.69)		Binary logistic regression model	Age, gender, and BMI, smoking, alcohol drinking, DM and HDL-C
Yun et al. 2016 (41)	USA	Hispanics	Cross-sectional	50/82 without NAFLD	NAFLD		USG		OR: 2.4(1.8–3.3)		Multivariate logistic regression model	
Kwak et al. 2015 (12)	China	East Asian	Cross-sectional	149/ 17612 without NAFLD	NAFLD		USG		OR: 1.35(1.03-1.77)		Multivariate logistic regression model	Age, sex, hypertension, diabetes, BMI, smoking, physical activity, total cholesterol, triglycerides and HDL-C cholesterol
Yue et al. 2019 (40)	Britain	Hispanics	Cross-sectional	772/10074 without NAFLD	NAFLD		USG		OR: 2.61(1.89-3.61)		Cox proportional hazard models	Age, sex, ethnicity, smoking and drinking status, SBP, total cholesterol, and HDL-C
Ruh et al. 2012 (38)	USA	Hispanics	Cross-sectional	265/ 12232 without NAFLD	NAFLD		USG		OR: 2.4(1.8-3.3)		Cox proportional hazard models	Age, sex, ethnicity, BMI, WC, diabetes, HDL-C, SBP, DBP, smoking, smoking, alcohol intake and physical activity
Carmen et al. 2009 (37)	USA	Hispanics	Prospective cohort	795/4307without NAFLD	NAFLD		USG		OR: 1.04(0.62-1.77)		Multivariate logistic regression model	Age, sex, education level, physical activity, total energy intake, hypertension, DM and BMI
Chang et al. 2018 (31)	Korea	East Asian	Prospective cohort	33506/94865 Men without NAFLD	NAFLD		USG		HR: 1.29(1.10-1.52)		Multivariate logistic regression model	Age, sex, BMI, smoking, alcohol intake, exercise, total calorie intake,
												and history of hypertension, history of diabetes, and medication for dyslipidemia
Chang et al. 2018 (31)	Korea	East Asian	Prospective cohort	15795/ 121830 Women without NAFLD	NAFLD		USG		HR: 1.05(0.86-1.28)		Multivariate logistic regression model	Age, sex, BMI, smoking, alcohol intake, exercise, total calorie intake,
												and history of hypertension, history of diabetes, and medication for dyslipidemia

GSD, gallstone disease; MetS, metabolic syndrome; NAFLD, nonalcoholic fatty liver disease; IR, insulin resistance; RR, relative ratio; OR: odds ratio; HR: hazard ratio; CI, confidence interval; BMI, body mass index; SBP, systolic blood pressure; DBP, diastolic blood pressure; WC, waist circumference; TG, triglyceride; FBG, fasting blood glucose; HDL-C, high density lipoprotein cholesterol; NCEP ATPIII, National Cholesterol Education Progrm Adult treatment Group III; CDS, Chinese Diabetes Society; USG, Ultrasonogram; DM, diabetes mellitus; HOMA-IR, homeostasis model of assessment-insulin resistance; eGFR, estimated glomerular filtration rate; GGT, γ-glutamyltransferase; GLO, serum globulins; ALB, serum albumin; BUN, blood urea nitrogen; GLU, total glucose.

There are six articles focusing on the relationship between GSD and NAFLD, two articles were prospective cohort studies, and four were cross-sectional studies. All patients were diagnosed with GS and NAFLD using Ultrasonogram (USG) Diagnosis. The total number of participants was as high as 322,630, including 215,497 patients with GSD, whose prevalence ranged from 4.44% to 76.66%, and 5926 patients with NAFLD, whose prevalence ranged from 1.86% to 34.43%. East Asians, Hispanics and Caucasians participated in four, one and one studies.

Six articles were included to explore the occurrence of NAFLD after cholecystectomy. A total of 261002 people participated, and the probability of suffering from NAFLD after the operation was as high as 60.97% Those who were East Asian or Hispanics engaged in just two and four studies respectively. Alcohol intake and physical activity were adjusted as covariates in three studies and only two study excluded patients with type 1 diabetes.

### 3.3 Quantitative analysis

#### 3.3.1 Risk of GSD on MetS occurrence

**Figure 3:**
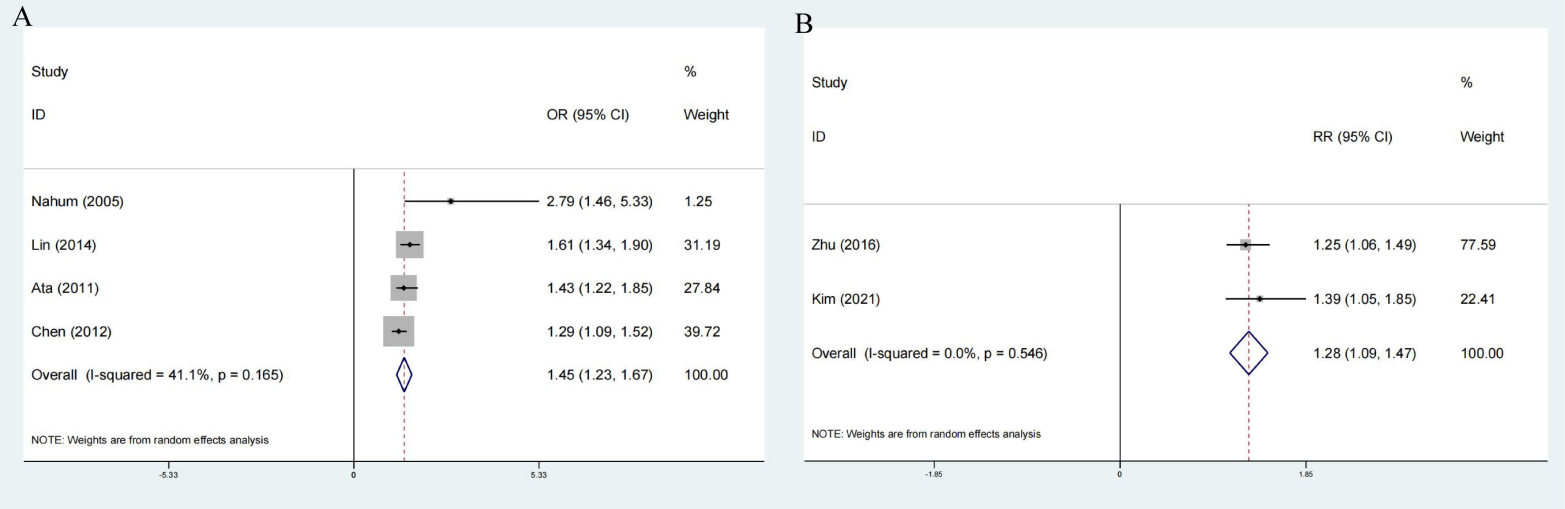
Forest plot on association between GSD and MetS **(A)** Pooled odds ratios of comparing the prevalence of MetS between GSD and non-GSD population (cross-sectional studies); **(B)** Pooled odds ratios comparing the prevalence of GSD between MetS and non-MetS population(cross-sectional studies); Pooled relative ratios of comparing the prevalence of GSD between NAFLD and non-NAFLD population; Pooled relative ratios of comparing the prevalence of NAFLD between GSD and non-GSD population. GSD, gallstone disease; MetS, metabolic syndrome; NAFLD, nonalcoholic fatty liver disease.

The pooled OR of incident MetS was 1.45 (95%CI: 1.23-1.67) with low heterogeneity (I^2^=41.1%, P=0.165; **Figure 3A**) for the yes versus no category of GSD in four enrolled cross-sectional studies.

##### 3.3.1.1 Subgroup, Sensitivity Analyses and Publication bias analysis

We attempt to assess the probable causes of heterogeneity using subgroup analysis due to the considerable variability in the overall study. Subgroup analysis was classified according to sample size, ethnicity, GSD incidence and calculation method. Among them, the subgroup analysis of GSD incidence, sample size and ethnicity could change heterogeneity. The merger OR of high GSD incidence is 1.76 (95%CI:0.62-2.90), which is the same as that of Westerners and low sample size (**Table 2**). Furthermore, the combined SMD of a single MetS component was specifically analyzed in four studies. Of the five components, hypertension was the only potential MetS component associated with increased prevalence of MetS. The combined SMD of 0.29 (95%CI:0.24-0.34) (**Figure 2**).

**Table 2:** Subgroup analysis assessing different variables for the risk of metabolic syndrome caused by gallstone disease in included cross-sectional studies.

			Heterogeneity	
Variables	Number	OR [95% Conf. Interval]	I-squared (%)	P
sample size				
<300	2	1.76 (0.62-2.90)	45.6%	0.175
>300	2	1.44 (1.12-1.75)	68.2%	0.076
P=0.165				
incidence rate(%)				
>15	2	1.76 (0.62-2.90)	45.6%	0.175
<15	2	1.44 (1.12-1.75)	68.2%	0.076
P<0.05				
Ethnicity				
Others (Caucasian, Hispanics)	2	1.76 (0.62-2.90)	45.6%	0.175
East Asian	2	1.44 (1.12-1.75)	68.2%	0.076
P=0.165				
calculation method				
Multivariate logistic regression	3	1.55 (1.31-1.78)	12.4%	0.319
Chi- Square test	1	1.29 (1.07-1.78)	NA	NA
P=0.165				

NA, Not applicable.

**Figure 4:**
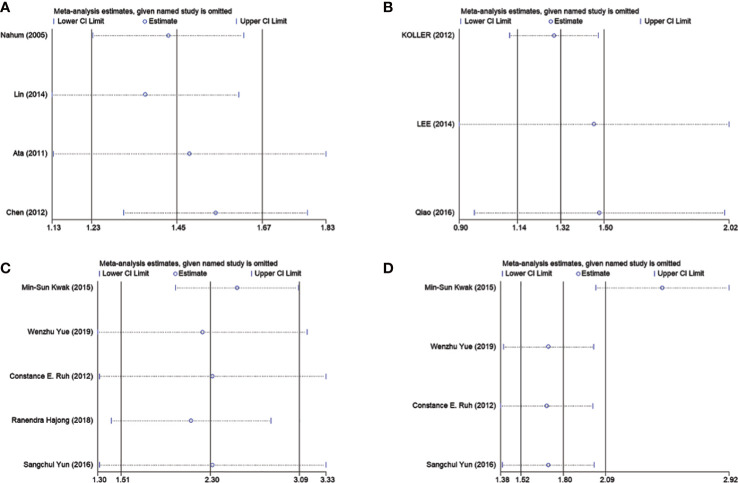
Sensitivity analyses of association between GSD and MetS as well as cholecystectomy and NAFLD. **(A)** Represents eliminated heterogeneity excluding a study of association between GSD and MetS in cross-sectional studies. **(B)** Represent eliminated heterogeneity excluding studies of association between GSD and NAFLD in cross-sectional studies. **(C, D)** Represent eliminated heterogeneity excluding studies of association between cholecystectomy and NAFLD in cross-sectional studies. GSD, gallstone disease; MetS, metabolic syndrome; NAFLD, nonalcoholic fatty liver disease.

In order to further explore the causes of overall heterogeneity, we carried out a sensitivity analysis. After omitting one study in turn and re-evaluating the summary OR of other studies, it is found that the heterogeneity is eliminated by excluding Chen’s study (**Figure 4A**). The larger sample size is the cause of this phenomenon.

**Figure 5:**
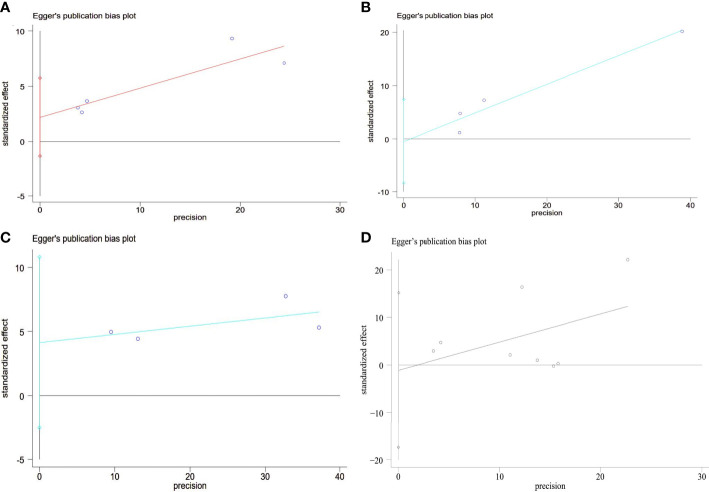
Egger’s funnel plot analysis of publication bias **(A)** Prevalence of MetS between GSD and non-GSD population, Egger’s test: P = 0.148. **(B)** Prevalence of GSD between NAFLD and non-NAFLD population, Egger’s test: P = 0.117. **(C)** Prevalence of NAFLD between GSD and non-GSD population, Egger’s test: P = 0.813. **(D)** Prevalence of NAFLD after cholecystectomy, Egger’s test: P = 0.873. GSD, gallstone disease; MetS, metabolic syndrome; NAFLD, nonalcoholic fatty liver disease.

Each study’s SE of the log OR was placed against the log OR for visual examination on the Egger’s funnel plot (**Figure 5A**). Egger’s test did not reveal any publication bias despite the funnel plot’s minor asymmetry (P = 0.148).

#### 3.3.2 Risk of MetS on GSD occurrence

The results of comprehensive analysis suggested that patients with MetS have an increased risk of developing GSD. The two studies included in the analysis are highly heterogeneous. (RR:1.28; 95%CI: 1.09-1.47; I^2^ = 0%, P=0.546) (**Figure 3B**).

##### 3.3.2.1 Dose-response analysis

**Figure 6:**
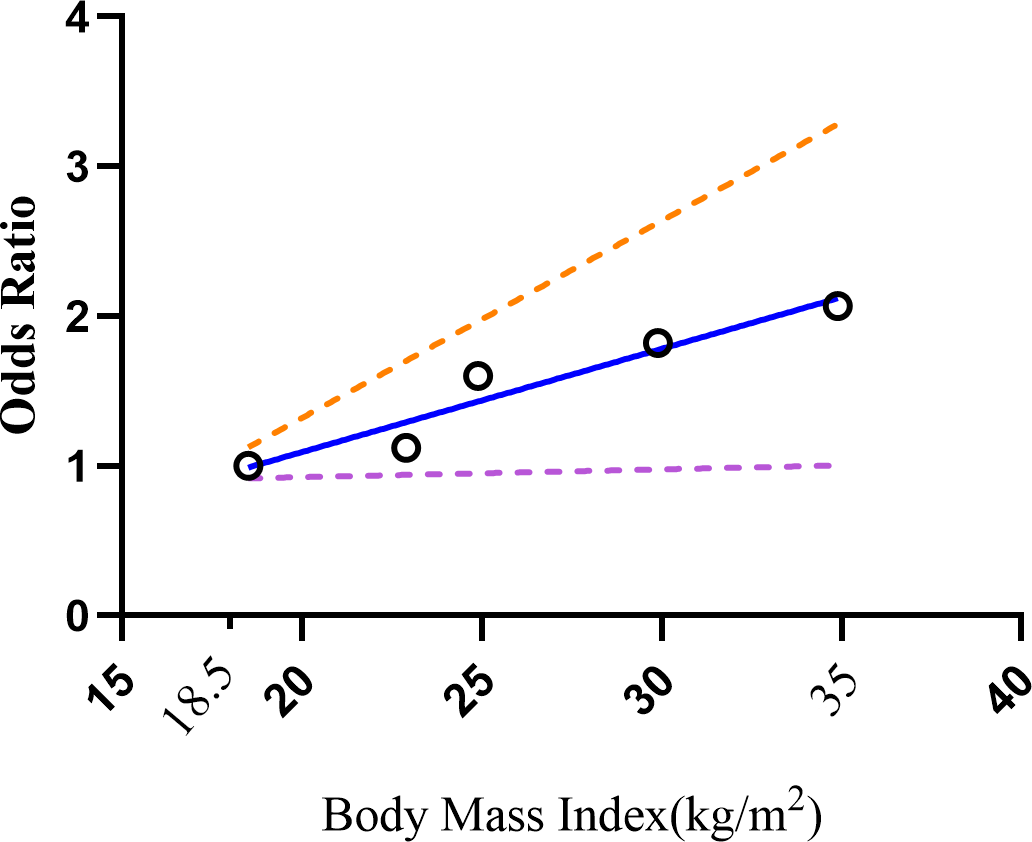
Dose-response relations between BMI levels and risk of GSD. GSD, gallstone disease; BMI, body mass index.

The original data of OR value and 95% confidence interval of GS disease in different intervals of BMI were given by Nahum et al. and Jonguk et al. When calculating and analyzing a single study, the results of both studies showed that the risk increase rate for every 1kg/m^2^ up in BMI was from 1% to 4%. We tried to combine the results of the two calculations and found that for every 1kg/m^2^ growth in BMI, the risk of GSD increased by 2%. If we specified the upper limit range of 35 and the lower limit range of 18.5, it could be seen that there is a positive linear relationship between them (P=0.0091, **Figure 6**).

#### 3.3.3 Risk of NAFLD on GSD occurrence

**Figure 7:**
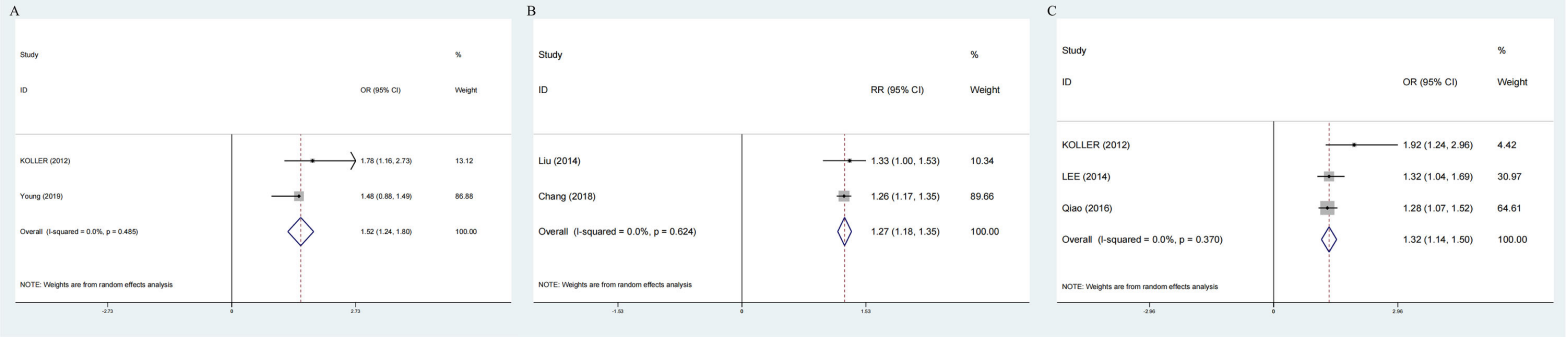
Forest plot on association between GSD and NAFLD. **(A)** Pooled odds ratios of comparing the prevalence of GSD between NAFLD and non-NAFLD population (cross-sectional studies); **(B)** Pooled relative ratios of comparing the prevalence of GSD between NAFLD and non-NAFLD population (prospective cohort studies); **(C)** Pooled relative ratios of comparing the prevalence of NAFLD between GSD and non-GSD population (cross-sectional studies). GSD, gallstone disease; NAFLD, nonalcoholic fatty liver disease.

Two prospective cohort and two cross-sectional studies reported the risk of NAFLD associated with elevated GSD. The pooled risk effects between groups with and without NAFLD was RR=1.27 (1.18-1.35) without heterogeneity, P=0.624 (**Figure 7B**) and OR=1.52 (95%CI: 1.24-1.80) without heterogeneity, P=0.485 (**Figure 7A**).

##### 3.3.3.1 Publication bias analysis

To assess the publication bias, Egger’s test was used. No significant publication bias was observed (Egger’s P = 0.813; **Figure 5B**).

#### 3.3.4 Risk of GSD on NAFLD occurrence

Three cross-sectional studies showed that NAFLD was associated with 1.3 times GSD risk (OR:1.32;95%CI:1.14-1.50), without heterogeneity, P=0.370 (**Figure 7C**).

*Subgroup, Sensitivity Analyses and Publication bias analysis* Subgroup analysis was classified by sample size, ethnicity, NAFLD incidence and calculation method. No subgroup caused significant heterogeneity (**Table 3**).

**Table 3:** Subgroup analysis assessing different variables for the risk of nonalcoholic fatty liver disease caused by gallstone disease in included cross-sectional studies.

					Heterogeneity	
Variable	Number		OR [95% Conf. Interval]		I-squared (%)	P
Ethnicity						
East Asian	2		1.29 (1.11-1.48)		0.00%	0.843
Northern Europe	1		1.92 (1.06-2.78)		NA	NA
P=0.0370						
sample size						
<8000	2		1.46 (0.92-2.02)		49.8%	0.158
>8000	1		1.32 (1.00-1.64)		NA	NA
P=0.370						
incidence rate						
<30	2		1,29 (1.11-1.48)		0.00%	0.843
>30	1		1.92 (1.06-2.78)		NA	NA
P=0.370						
calculation method						
multivariate logistic regression	2		1.46 (0.92-2.02)		49.8%	0.03
Binary logistic regression	1		1.32 (1.00-1.64)		NA	NA
P=0.370						

NA, Not applicable.

We did sensitivity analysis by deleting one research at a time from the meta-analysis. Koller et al. ‘s study could significantly change the OR and heterogeneity of the aggregate. It may be related to the low sample size (**Figure 4B**). Use of Egger’s test was made. There was no evidence of publication bias (P =0.873; **Figure 5C**).

#### 3.3.5 Risk of NAFLD after cholecystectomy

**Figure 8:**
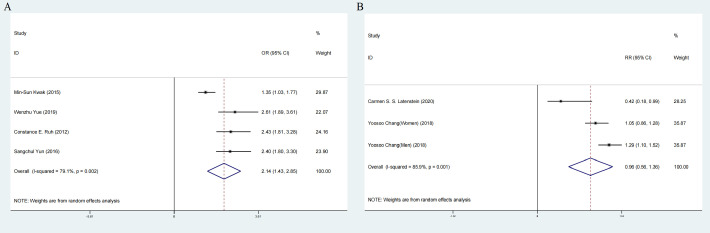
Forest plot of association between cholecystectomy and NAFLD. **(A)** Pooled odds ratios of comparing the prevalence of NAFLD between cholecystectomy and non-cholecystectomy population (cross-sectional studies). **(B)** Pooled odds ratios of comparing the prevalence of NAFLD between cholecystectomy and non-cholecystectomy population (prospective cohort studies).NAFLD, nonalcoholic fatty liver disease.

Comprehensive analysis of all cross-sectional literature manifested that the risk of NAFLD events after cholecystectomy was higher than that without cholecystectomy intervention (OR:2.14;95%CI:1.43-2.85), with high heterogeneity (I^2^=79.1%, P < 0.05; **Figure 8A**). But the prospective cohort literature indicated an opposite result (RR:0.96,95%CI:0.56-1.36) (**Figure 8B**).

##### 3.3.5.1 Subgroup, meta-regression analyses, Sensitivity Analyses and Publication bias analysis

Subcomponent analysis included ethnicity, sample size, adjusted alcohol intake, adjusted physical activity, BMI and Excluded 1 diabetes in cross-sectional study. Hispanics, BMI>25kg/m^2^, adjusted physical activity, excluded 1diabetes and adjusted alcohol intake lowered the heterogeneity (**Table 4**).

**Table 4:** Subgroup analysis assessing different variables for the risk of nonalcoholic fatty liver disease after cholecystectomy in included cross-sectional studies.

						Heterogeneity	
Variables			Number	OR [95% Conf. Interval]		I-squared (%)	P
BMI							
<25			2	1.82 (0.80-2.85)		83.5%	0.014
>25			2	2.51 (1.95-3.06)		0.00%	<0.05
P <0.05							
ethnicity							
Asian			1	1.35 (0.98-1.72)		NA	NA
Hispanics			4	2.47 (2.02-2.92)		0.00%	0.929
P <0.05							
Sample size							
<10000			1	2.40 (1.65-3.15)		NA	NA
>10000			3	2.07 (1.18-2.96)		82.7%	<0.05
P <0.05							
Adjusted-alcohol intake							
No			1	1.35 (0.98-1.72)		NA	NA
Yes			3	2.47 (2.02-2.92)		0.00%	0.929
P <0.05							
Adjusted-Physical activity							
No			2	2.49 (1.93-3.06)		0.00%	0.718
Yes			2	1.84 (0.79-2.90)		84.9%	<0.05
P <0.05							
Excluded 1 diabetes							
No			2	2.49 (1.93-3.06)		0.00%	0.718
Yes			2	1.84 (0.79-2.90)		84.9%	<0.05
P <0.05							

NA, Not applicable.

**Figure 9:**
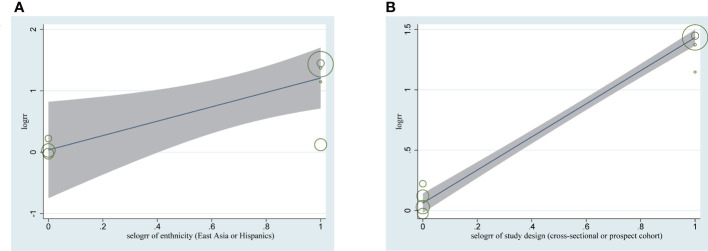
Meta-regression analyses assessing the heterogeneity of between cholecystectomy and NAFLD **(A)** Impact of ethnicity on associations between NAFLD between cholecystectomy. **(B)** Impact of study design on associations between NAFLD and cholecystectomy. NAFLD, nonalcoholic fatty liver disease.

Meta-regression analyses showed that study design and ethnicity were the two causes of high heterogeneity (P < 0.05) (**Figure 9**)

Sensitivity analysis showed that the heterogeneity of cross-sectional study was affected by Kwak et al.’s study and Carmen et al. ‘s study could significantly change the RR and heterogeneity of the aggregate (**Figures 4C, D**). It may be related to the high sample size. Egger’s test was used. No significant publication bias was observed (P =0.873; **Figure 5D**).

## 4 Discussion

Long-term research was conducted on both the MetS/NAFLD and GSD connection, as well as the relationship between cholecystectomy and NAFLD. The purpose of this meta-analysis was to conduct a complete examination of all the currently available data and to integrate that information to arrive at conclusive findings about this possible link. There were two primary outcomes. Firstly, there was a complex association between GSD and metabolic disorders including NAFLD and MetS. Secondly, NAFLD was probably related to cholecystectomy. After we performed subgroup analyses, meta-regression analyses and dose-response analyses, some new results were obtained to support the primary outcomes. To be specific, Hypertension may increase the incidence of GSD. And approximate 2% increment was observed on the GSD incidence per 1 kg/m^2^ of BMI elevation. Furthermore, obese GSD patients who have undergone cholecystectomy were more likely to develop NAFLD than non-obese GSD patients.

After pooling previous studies, we found that MetS could cause GSD (without heterogeneity). And GSD was a risk factor of MetS with low heterogeneity. However, we still performed subgroup analyses and the results showed that the heterogeneity was strengthened by sample size, GSD incidence and ethnicity. So we supposed that the epidemiological evidence of MetS could make sense. Prevalence among white adults in developed countries was as high as 10% to 15% (52). And The National Health and Nutrition Examination Survey in the United States pointed to an overall MetS prevalence of 23.7% (53). Nevertheless, the popularity of GSD in China did not exceed 15% (54). By 2000, the prevalence of MetS was 15.1% (54), significantly lower than that in the United States. The results of the subgroup analysis did not confirmed that Hispanics and Caucasians could affect MetS incidence and we think it was caused by low enrolled population.

A liver condition known as nonalcoholic fatty liver disease (NAFLD) can range from moderate hepatic steatosis to nonalcoholic steatohepatitis (NASH) (55). NASH can subsequently develop into advanced liver fibrosis, cirrhosis, or hepatocellular carcinoma (55). The prevalence of NAFLD in the general population worldwide is as high as 20% (56). We found that NAFLD patients were 15 times more likely to develop GSD than non-NAFLD patients (without heterogeneity), whereas GSD patients were 1.3 times more likely to develop NAFLD than non-GSD patients (without heterogeneity). Some studies have demonstrated a bidirectional and independent association between GSD and NAFLD (57). A longitudinal cohort Asian study followed 11200 participants for 6 years and found NAFLD was an independent risk factor for GSD (RR=1.2381, 95%CI:1.003-1.528), especially in women (RR=1.707,95CI%: 1.245-2.341) (34). Similar results were observed by Loria et al. (58) in a cohort with a greater frequency of GSD than the general community. Despite adjusting the factors significantly related to GSD for NAFLD patients in their investigation, Yilmaz et al. (59) could not find a correlation between the diagnosis of GSD and nonalcoholic steatohepatitis. (OR=1.03; 95% CI 0.5-2.1), indicating that GSD was not an independent risk factor for NAFLD. However, a “chicken and egg” dispute is now going on over the temporal association between NAFLD and GSD, and there is no explicit agreement on the topic (60). Our results only demonstrated a temporal association of NAFLD affecting GSD.

Given the analysis of individual MetS components, only high systolic blood pressure was significantly associated with high GSD prevalence and it raised the heterogeneity (**Figure 2**). Perhaps due to insufficient data in the included studies, it was inconsistent with previous meta-analyses that all components of the metabolic syndrome were positively associated with GSD prevalence (43). Recently, Zhang et al. (61)conducted a cross-sectional research in a Chinese population from Liaoning Province with the purpose of examining the effects of systolic and diastolic blood pressure on GSD. They achieved findings that were comparable with ours. As one of the diagnostic criteria for MetS, its association with gallstones can be explained by insulin resistance (62). Worsening insulin resistance can trigger mechanisms that increase renal sodium reabsorption and sympathetic nervous system activity (62, 63), ultimately leading to hypertension in patients with MetS. In addition, high blood pressure may also be inseparable from obesity according to our results. Liew et al. (55) put forward that Asian obese patients had higher diastolic blood pressure with cholelithiasis. But the mechanism is unclear, and perhaps it is related to insulin resistance. Furthermore, Hsu et al. (64)discovered that obesity represented by high waist circumference and BMI is the main risk factor for GSD. But few studies compared its detailed degree of influence. We hypothesized that BMI might be a useful marker for predicting and screening for GSD based on the positive linear dose-response relationship. It is well known that BMI is a specific parameter for overweight and obesity (65). On the one hand, high BMI incurred larger gallbladder and higher cholesterol synthase activity (66). On the other hand, mature adipocytes, a bridge between obesity and GSD, could secrete leptin (67). Such fat factor played an irreplaceable role in regulating the motility of gallbladder (GB) (68) and promoting the secretory function of stone formation (69). For this, obese patients often had the phenomenon of insufficient contractile ability of GB and supersaturated cholesterol in bile (69). And IR could promote stone formation in normal and overweight people (70). It is worth noting that obesity related to MetS was more about highlighting abdominal obesity caused by high waists (71). Tsai et al. (71) proved that the abdominal circumference and waist-to-hip ratio are related with an increased risk of cholecystectomy, irrespective of BMI in Western women. For men, using BMI alone may mask excess fat (72). And it is easier to measure waist circumference than BMI. However, none of the registered studies reported a dose-response of high waist circumference in our meta-analysis. Accordingly, it is essential to strengthen the study on the incidence of GSD in the degree of high waist circumference.

NAFLD has traditionally been considered the hepatic manifestation of the metabolic syndrome because NAFLD is often associated with repertoire of MetS features (73). Leite et al. (74) found that about two-thirds of obese and type 2 diabetic patients had hepatic steatosis. About 50% of patients with hyperlipidemia (75) and 50% of patients with essential hypertension (76) also had hepatic steatosis. That’s why experts emphasized changing NAFLD to MAFLD in recent years (77). MAFLD more accurately reflects the current understanding of fatty liver disease associated with metabolic dysfunction (77). According to the findings of epidemiological research, the rise in the prevalence of obesity was the primary cause of the increase in the death rate from NAFLD (78). Although the increased incidence of NAFLD was often attributed to the obesity epidemic, NAFLD was detected in non-obese individuals (79). So it is a more complex disease process. The relationship between abnormal glucose metabolism and fatty liver disease has been agreed upon (36). Fasting blood glucose levels were proven to be wholly associated with the presence of gallstones in NAFLD patients in a research comparing those with simple NAFLD to those with NAFLD complicated by GSD (36). That is, NAFLD might promote GSD through metabolic syndrome factors. However, Lu et al. (80) highlighted that type 2 diabetes mellitus (T2DM) predisposed to GSD more than NAFLD. Meanwhile, T2DM can aggravate the course of NAFLD (81). Therefore, the diagnosis and treatment of NAFLD cannot be ignored in patients with both diabetes and GSD.

A growing number of studies suggested in multivariate adjustment analyses that gallstones were no longer independently associated with NAFLD but cholecystectomy was the independent risk factor for NAFLD (12, 42). Though the combined results of the cross-sectional studies showed cholecystectomy is related to NAFLD but the heterogeneity was high. Furthermore, we can’t confirm their causality after combining from the results from prospective studies. Otherwise, potential defects of these two enrolled prospective studies (31, 37) on GSD and NAFLD should be considered. To be specific, Carmen et al. only mentioned one ultrasound during the follow up which was not qualified to prove that the NAFLD is posterior to the surgery because at least two ultrasounds are necessary. And Chang et al. showed a slight independent relationship between GSD and NAFLD in their multivariate analysis. But this association was only observed in males but not in females. All in all, more prospective studies are worthy on further investigation to explore whether cholecystectomy could cause NAFLD independently.

High heterogeneity can be caused by racial factors. According to our subgroup analysis and regression analysis, the phenomenon in Hispanics is about twice as common as than risks in Asians. Global figures showed that while NAFLD prevalence in Asia is only 27% (82), it is approximately 30% in the United States (83). According to research by Golabi et al. (84), the prevalence of NAFLD among Asian American adults was almost three times lower than it is among Hispanic Americans (47 vs. 26%, respectively). And obesity also might be a reasonable explanation for potential heterogeneity. We discovered that obese patients with gallstones had a greater chance of developing NAFLD following cholecystectomy than non-obese patients did when we compared the degree of BMI as a confounding variable. Non-obesity individuals did not have gallstones (**Figure 8B**). It suggested that cholecystectomy may aggravate the disorder of lipid distribution in some way and promote the accumulation of fat in the liver. The fact that Hispanic patients who undergo non-obesity cholecystectomy had significantly higher levels of NAFLD than non-Hispanic patients supports this conclusion (85). A study by Amigo et al. (86) found that cholecystectomy in mice increased bile cholesterol and energy consumption, leading to an increase in triglyceride and very low density lipoprotein levels and worsening NAFLD in mice. It provided strong evidence for the effect of cholecystectomy on lipid metabolism (86). In addition, Kakati et al. (87) found that the median time to diagnosis of NAFLD after cholecystectomy was approximately 6.2 years. But the timing of cholecystectomy was not associated with disease progression in patients with preoperatively diagnosed NAFLD (87). It indicated that it was indispensable to check NAFLD regularly after cholecystectomy. And in the future, more clinical studies should be put into this direction.

**Figure 10:**
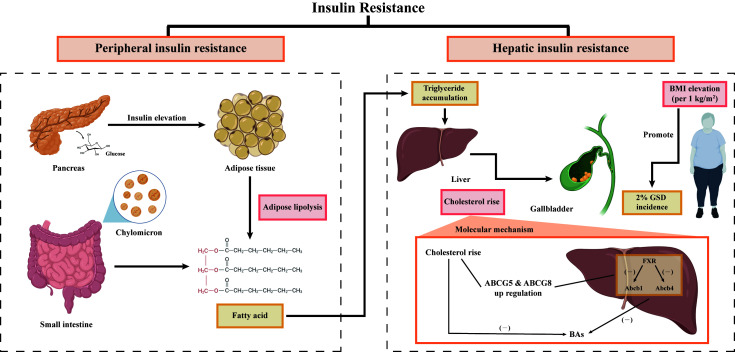
Potential mechanisms between GSD and IR. GSD, gallstone disease; BMI, body mass index; FXR, farnesoid X receptor.

In fact, IR provides a key link between MetS, NAFLD, increased susceptibility to gallstones, and cholecystectomy (88). The core of lipid metabolism disorder is insulin resistance (89). Lipolysis could be induced by peripheral insulin resistance (90). A large amount of free fatty acids (FFA) entered the liver from the peripheral tissue to produce more fat (89). Meanwhile, lowering activity of the peripheral lipoprotein lipase predisposed an increase in chylomicron (89). The process affected the regulation of triglycerides in the liver, resulting in the accumulation of triglycerides, which further aggravated liver insulin resistance (89). The increase of triglycerides accelerated the synthesis of endogenous cholesterol. It may have something to do with the obesity (91). However, Scragg et al. (92) explained that the phenomenon that the increase of plasma insulin concentration aggravates the incidence of GSD is independent of obesity but is related to women and age. At present, some researchers also showed that obesity is neither necessary nor sufficient for the pathogenesis of GSD (18). Therefore, there may be an indirect relationship between obesity and insulin resistance to regulate GSD jointly. Other studies had explained the molecular mechanism (93, 94). When the liver develops insulin resistance on its own, the nuclear heterodimeric receptor farnesoid X receptor (FXR) gene was down-regulated and the corresponding receptor expression decreased (93). And then bile acid transporter protein Abcb11 and phospholipid transporter Abcb4 reduced (93). Finally, bile acid could not be transferred and accumulated, and the content of bile acid in bile decreased (93). In another pathway, up-regulated cholesterol secretion genes ABCG5 and ABCG8 promoted more expression of cholesterol transporters and finally increased cholesterol secretion (94). Inhibition of 7α -hydroxylase contributed to the conversion of cholesterol to bile acids, resulting in cholesterol supersaturation (95). Combined with our data, **Figure 10** summarizes and quantitatively demonstrates the underlying mechanisms of IR and GSD association.

It is important to note the reliability and usefulness of our findings. We evaluated the association between GSD and NAFLD/MetS by consideration of risk direction. To be specific, the pooled GSD risk in subjects with/without NAFLD/MetS, and risk of metabolic derangements based on GSD status were evaluated respectively in different models. Based on the positive linear dose-response relationship, using BMI to predict GSD has a high cost-effectiveness (64). In the future, a perfect and standardized prediction model (96) can be made for clinical use and even help people to perform self-prevention. This model allows patients to compare their risk of GSD based on BMI measurements when they are in the hospital or at home. Weight loss treatments, such as more activity and a restricted diet, should be used in patients with higher risk of GSD (97). Due to the potential causal relationship between IR and GSD, reducing modifiable risk factors for MetS and NAFLD is expected to be a future target for drug design (98). For example, try coming up with some medicines that can boost transporter efficiency and enzyme activity (98). We found that obesity may increase the incidence of NAFLD in GSD patients who have undergone cholecystectomy. So we presumed that subsequent studies should investigate whether GB-preserving cholecystolithotomy is preferable to cholecystectomy from the standpoint of metabolic regulation (99). And it would improve the prognosis, reduce the risk of postoperative complications, and lower the cost of medical insurance.

We noted that there were certain limitations even though the majority of the included research were of high quality. First of all, we were unable to conclude with certainty that the observed outcomes were not attributable to NAFLD/MetS itself or to any of the other possible confounding variables. Second, only a small number of prospective cohort studies were included in each analysis, which limited the ability to obtain more conclusive evidence and the conclusions need to be confirmed in more and larger cohort studies. Third, it is necessary to state the drawbacks of combining data from different recruited research, such as the lack of consistency in risk measurements and statistical methodologies. RR and OR from different statistical models exhibited discrepant meaning, suggesting the presence of heterogeneities if the two were combined. Fourth, very few included studies could support the dose-response analysis of BMI, so the relationship between BMI and GSD would be compared later. Fifth, this study is not a mechanism study, so the potential mechanism of IR affecting GSD has not been well described. We will follow up on animal experiments to explore this process. Sixth, due to the lack of data on relevant risk factors provided in the registered articles, we cannot probe into the detailed biological interaction between NAFLD and BMI after cholecystectomy. Therefore, we are planning to collect more information based on information from our center to evaluate the quantitative relationship between cholecystectomy and NAFLD incidence in patients with different BMI categories (100, 101). In addition, omic data played crucial roles in exploring the mechanism of complex disease (102). And multi-omics data was confirmed to disclose the function of genes based on network analysis (103). Actually, the temporal relationship between GSD and NAFLD is an interesting study topic and we are planning to clarify this causal-effect interaction between these two covariates based on cohort study. Currently, we are collecting gallbladder samples from sample who received cholecystectomy which might provide more reliable evidence to reveal the mechanism of complex associations between GSD and insulin resistance.

## 5 Conclusion

This meta-analysis provided evidence that the close relationship between GSD and MetS/NAFLD, or insulin resistance, and the close relationship between cholecystectomy and NAFLD. No matter what kind of disease, geographical differences in the risks are greater in the America, compared to Europe and Asia. We also observed that calculating BMI might be a useful and customized technique for determining the likelihood of developing GSD. Paying attention to the control of blood pressure and blood sugar is helpful in alleviating GSD. In the future, well-designed and high-quality prospective studies are needed to confirm these effects and to further study cholecystolithotomy through metabonomics.

## Data availability statement

The original contributions presented in the study are included in the article/**Supplementary Material**. Further inquiries can be directed to the corresponding author.

## Author contributions

ZL conceived and designed the study. JL and QL performed experiment and extracted information. JL and QL analyzed the data. JL wrote the manuscript. ZL reviewed the manuscript. ZL provided the funding support. All authors contributed to the article and approved the submitted version.

## Funding

This study is supported by Innovative Research Groups of National Natural Science Foundation of China (81721091), Major program of National Natural Science Foundation of China (91542205), National S&T Major Project (2017ZX10203205), National Natural Science Foundation of China (81902813), Zhejiang International Science and Technology Cooperation Project (2016C04003), Zhejiang Provincial Natural Science Foundation of China (LY22H030008), Zhejiang Medical Association (2019ZYC-A81), International Youth Exchange Programme by China Association for Science and Technology (2019), Tianqing Liver Diseases Research Fund (TQGB20200114), Organ Transplantation Overseas Training for Youth Talents from Shulan Excellent Talent Project, CSCO (Chinese Society Of Clinical Oncology)-Bayer Tumor Research Funding (Y-bayer202001/zb-0003), Chen Xiao-ping Foundation for the Development of Science and Technology of Hubei Province (CXPJJH122002-078), Beijing iGandan Foundation (1082022-RGG022), Open Fund of Key laboratory of High-Incidence-Tumor Prevention & Treatment (Guangxi Medical University), Ministry of Education, and Xinmiao Talent Supporting Program (2022R421A031).

## Acknowledgments

We profoundly grateful to our supervisor, ZL, whose illuminating instruction and expert advice have guided me through every step of my writing of this research. Our great gratitude also goes to some of our friends and classmates who have selfless and generously helped me with my thesis.

## Conflict of interest

The authors declare that the research was conducted in the absence of any commercial or financial relationships that could be construed as a potential conflict of interest.

## Publisher’s note

All claims expressed in this article are solely those of the authors and do not necessarily represent those of their affiliated organizations, or those of the publisher, the editors and the reviewers. Any product that may be evaluated in this article, or claim that may be made by its manufacturer, is not guaranteed or endorsed by the publisher.

## Abbreviations

BMI, body mass index; CDS, Chinese Diabetes Society; CI, confidence interval; FFA, free fatty acids; FXR, farnesoid X receptor; GB, gallbladder; GSD, gallstone disease; HR: hazard ratio; IR, insulin resistance; WOS, Web of Science; MetS: metabolic syndrome; NAFLD, nonalcoholic fatty liver disease; NASH, nonalcoholic steatohepatitis; NCEP ATPIII, National Cholesterol Education Program Adult Treatment Group III; NOS, Newcastle-Ottawa Scale; OR, odds ratio; RR, risk ratio; SMD, standardized mean differences; T2DM, type 2 diabetes mellitus; USG, Ultrasonogram.
