# Supplementary material for: Complex impacts of gallstone disease on metabolic syndrome and nonalcoholic fatty liver disease
Source: Front Endocrinol (Lausanne). 2022 Nov 23;13:1032557. doi: 10.3389/fendo.2022.1032557 (PMC9727379; doi:10.3389/fendo.2022.1032557)
Supplement: Supplementary file 1 [file DataSheet_1.docx]

Supplementary Material

**PRISMA 2020 flow diagram**

**Identification of studies via databases and registers**

Records removed *before screening*:

Duplicate records removed

(n = 894)

Records marked as ineligible by automation tools (n = 0)

Records removed for other reasons (n = 0)

Records identified from:

Databases (n = 4104):

PubMed(n=174)

Embase(n=2009)

ISI(n=1921)

Registers (n = 0)

**Identification**

Records screened

(n =3210)

Records excluded

(n = 3123)

**Screening**

Reports sought for retrieval

(n =87)

Reports not retrieved

(n = 0)

Reports excluded:

No subjects of interest (n =28)

No outcome of interest (n =30)

No specific MetS definition (n =3)

Focused on GSD-IR association (n=6)

Focused on cholecystectomy-NASH (n=1)

Reports assessed for eligibility

(n =87)

Studies included in review

(n = 19)

**Included**

*From:*  Page MJ, McKenzie JE, Bossuyt PM, Boutron I, Hoffmann TC, Mulrow CD, et al. The PRISMA 2020 statement: an updated guideline for reporting systematic reviews. BMJ 2021;372:n71. doi: 10.1136/bmj.n71

For more information, visit: <http://www.prisma-statement.org/>


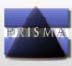
**PRISMA 2020 for Abstracts Checklist**

| **Section and Topic** | **Item #** | **Checklist item** | **Reported (Yes/No)** |
| --- | --- | --- | --- |
| **TITLE** | | |  |
| Title | 1 | Identify the report as a systematic review. | Yes |
| **BACKGROUND** | | |  |
| Objectives | 2 | Provide an explicit statement of the main objective(s) or question(s) the review addresses. | Yes |
| **METHODS** | | |  |
| Eligibility criteria | 3 | Specify the inclusion and exclusion criteria for the review. | Yes |
| Information sources | 4 | Specify the information sources (e.g. databases, registers) used to identify studies and the date when each was last searched. | Yes |
| Risk of bias | 5 | Specify the methods used to assess risk of bias in the included studies. | Yes |
| Synthesis of results | 6 | Specify the methods used to present and synthesise results. | Yes |
| **RESULTS** | | |  |
| Included studies | 7 | Give the total number of included studies and participants and summarise relevant characteristics of studies. | Yes |
| Synthesis of results | 8 | Present results for main outcomes, preferably indicating the number of included studies and participants for each. If meta-analysis was done, report the summary estimate and confidence/credible interval. If comparing groups, indicate the direction of the effect (i.e. which group is favoured). | Yes |
| **DISCUSSION** | | |  |
| Limitations of evidence | 9 | Provide a brief summary of the limitations of the evidence included in the review (e.g. study risk of bias, inconsistency and imprecision). | Yes |
| Interpretation | 10 | Provide a general interpretation of the results and important implications. | Yes |
| **OTHER** | | |  |
| Funding | 11 | Specify the primary source of funding for the review. | Yes |
| Registration | 12 | Provide the register name and registration number. | No |

*From:*  Page MJ, McKenzie JE, Bossuyt PM, Boutron I, Hoffmann TC, Mulrow CD, et al. The PRISMA 2020 statement: an updated guideline for reporting systematic reviews. BMJ 2021;372:n71. doi: 10.1136/bmj.n71

For more information, visit: <http://www.prisma-statement.org/>


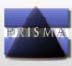
**PRISMA 2020 Checklist**

| **Section and Topic** | **Item #** | **Checklist item** | **Location where item is reported** |
| --- | --- | --- | --- |
| **TITLE** | | |  |
| Title | 1 | Identify the report as a systematic review. | 1 |
| **ABSTRACT** | | |  |
| Abstract | 2 | See the PRISMA 2020 for Abstracts checklist. | 2 |
| **INTRODUCTION** | | |  |
| Rationale | 3 | Describe the rationale for the review in the context of existing knowledge. | 3 |
| Objectives | 4 | Provide an explicit statement of the objective(s) or question(s) the review addresses. | 3-4 |
| **METHODS** | | |  |
| Eligibility criteria | 5 | Specify the inclusion and exclusion criteria for the review and how studies were grouped for the syntheses. | 5 |
| Information sources | 6 | Specify all databases, registers, websites, organisations, reference lists and other sources searched or consulted to identify studies. Specify the date when each source was last searched or consulted. | 5 |
| Search strategy | 7 | Present the full search strategies for all databases, registers and websites, including any filters and limits used. | 5 |
| Selection process | 8 | Specify the methods used to decide whether a study met the inclusion criteria of the review, including how many reviewers screened each record and each report retrieved, whether they worked independently, and if applicable, details of automation tools used in the process. | 5 |
| Data collection process | 9 | Specify the methods used to collect data from reports, including how many reviewers collected data from each report, whether they worked independently, any processes for obtaining or confirming data from study investigators, and if applicable, details of automation tools used in the process. | 5 |
| Data items | 10a | List and define all outcomes for which data were sought. Specify whether all results that were compatible with each outcome domain in each study were sought (e.g. for all measures, time points, analyses), and if not, the methods used to decide which results to collect. | 5 |
|  | 10b | List and define all other variables for which data were sought (e.g. participant and intervention characteristics, funding sources). Describe any assumptions made about any missing or unclear information. | 5 |
| Study risk of bias assessment | 11 | Specify the methods used to assess risk of bias in the included studies, including details of the tool(s) used, how many reviewers assessed each study and whether they worked independently, and if applicable, details of automation tools used in the process. | 6 |
| Effect measures | 12 | Specify for each outcome the effect measure(s) (e.g. risk ratio, mean difference) used in the synthesis or presentation of results. | 6 |
| Synthesis methods | 13a | Describe the processes used to decide which studies were eligible for each synthesis (e.g. tabulating the study intervention characteristics and comparing against the planned groups for each synthesis (item #5)). | 6 |
|  | 13b | Describe any methods required to prepare the data for presentation or synthesis, such as handling of missing summary statistics, or data conversions. | 6 |
|  | 13c | Describe any methods used to tabulate or visually display results of individual studies and syntheses. | 6 |
|  | 13d | Describe any methods used to synthesize results and provide a rationale for the choice(s). If meta-analysis was performed, describe the model(s), method(s) to identify the presence and extent of statistical heterogeneity, and software package(s) used. | 6 |
|  | 13e | Describe any methods used to explore possible causes of heterogeneity among study results (e.g. subgroup analysis, meta-regression). | 6 |
|  | 13f | Describe any sensitivity analyses conducted to assess robustness of the synthesized results. | 6 |
| Reporting bias assessment | 14 | Describe any methods used to assess risk of bias due to missing results in a synthesis (arising from reporting biases). | 6 |
| Certainty assessment | 15 | Describe any methods used to assess certainty (or confidence) in the body of evidence for an outcome. | 6 |
| **RESULTS** | | |  |
| Study selection | 16a | Describe the results of the search and selection process, from the number of records identified in the search to the number of studies included in the review, ideally using a flow diagram. | 7 |
|  | 16b | Cite studies that might appear to meet the inclusion criteria, but which were excluded, and explain why they were excluded. | 7 |
| Study characteristics | 17 | Cite each included study and present its characteristics. | 7 |
| Risk of bias in studies | 18 | Present assessments of risk of bias for each included study. | 8 |
| Results of individual studies | 19 | For all outcomes, present, for each study: (a) summary statistics for each group (where appropriate) and (b) an effect estimate and its precision (e.g. confidence/credible interval), ideally using structured tables or plots. | 8-10 |
| Results of syntheses | 20a | For each synthesis, briefly summarise the characteristics and risk of bias among contributing studies. | 8-10 |
|  | 20b | Present results of all statistical syntheses conducted. If meta-analysis was done, present for each the summary estimate and its precision (e.g. confidence/credible interval) and measures of statistical heterogeneity. If comparing groups, describe the direction of the effect. | 8-10 |
|  | 20c | Present results of all investigations of possible causes of heterogeneity among study results. | 8-10 |
|  | 20d | Present results of all sensitivity analyses conducted to assess the robustness of the synthesized results. | 8-10 |
| Reporting biases | 21 | Present assessments of risk of bias due to missing results (arising from reporting biases) for each synthesis assessed. | 9-10 |
| Certainty of evidence | 22 | Present assessments of certainty (or confidence) in the body of evidence for each outcome assessed. | 9-10 |
| **DISCUSSION** | | |  |
| Discussion | 23a | Provide a general interpretation of the results in the context of other evidence. | 11-13 |
|  | 23b | Discuss any limitations of the evidence included in the review. | 14 |
|  | 23c | Discuss any limitations of the review processes used. | 14 |
|  | 23d | Discuss implications of the results for practice, policy, and future research. | 14 |
| **OTHER INFORMATION** | | |  |
| Registration and protocol | 24a | Provide registration information for the review, including register name and registration number, or state that the review was not registered. | 5 |
|  | 24b | Indicate where the review protocol can be accessed, or state that a protocol was not prepared. | 5 |
|  | 24c | Describe and explain any amendments to information provided at registration or in the protocol. | 5 |
| Support | 25 | Describe sources of financial or non-financial support for the review, and the role of the funders or sponsors in the review. | 18 |
| Competing interests | 26 | Declare any competing interests of review authors. | 18 |

*From:*  Page MJ, McKenzie JE, Bossuyt PM, Boutron I, Hoffmann TC, Mulrow CD, et al. The PRISMA 2020 statement: an updated guideline for reporting systematic reviews. BMJ 2021;372:n71. doi: 10.1136/bmj.n71

For more information, visit: <http://www.prisma-statement.org/>

FigureS1. study flow diagram


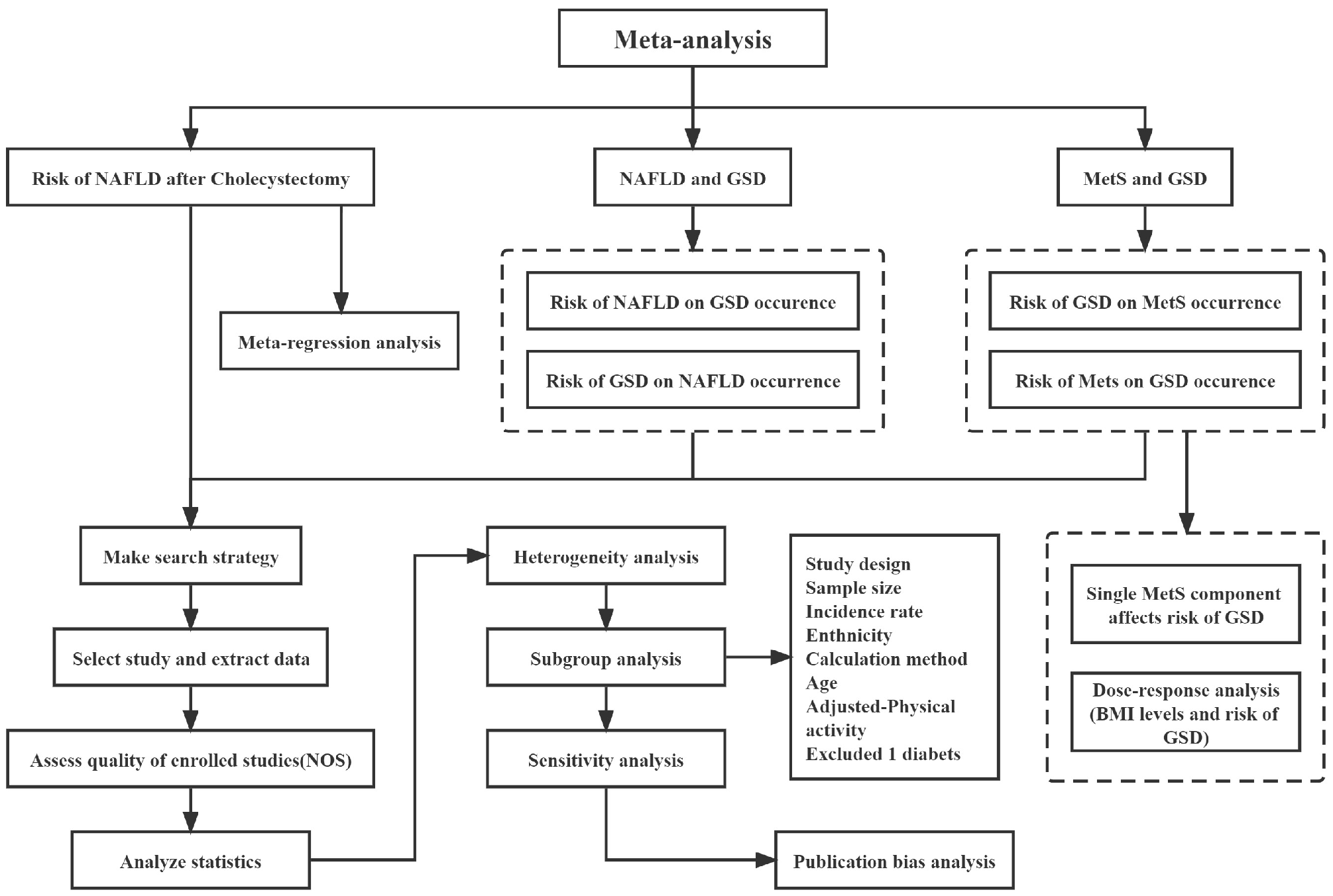


Abbreviations: GSD, gallstone disease; MetS, metabolic syndrome; NAFLD, nonalcoholic fatty liver disease.

FigureS2. GRADE Summary of Findings


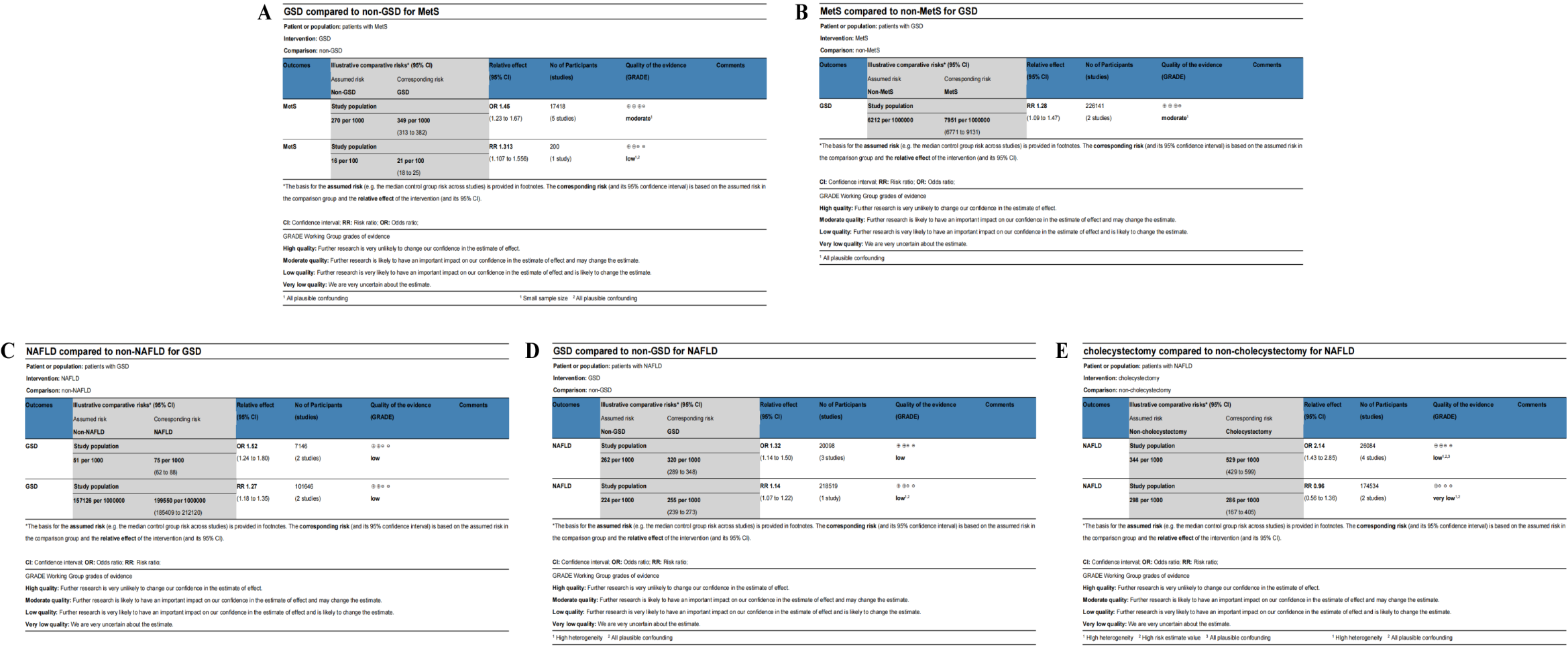


**(A)** The summary of findings for GSD compared to non-GSD for MetS including cross-sectional study and prospective cohort study; **(B)** The summary of findings for MetS compared to non-MetS for GSD including prospective cohort study; **(C)** The summary of findings for NAFLD compared to non-NAFLD for GSD including cross-sectional study and prospective cohort study; **(D)** The summary of findings for GSD compared to non-GSD for NAFLD including cross-sectional study and prospective cohort study; **(E)** The summary of findings for cholecystectomy compared to non-cholecystectomy for NAFLD including cross-sectional study and prospective cohort study.

Abbreviations: GSD, gallstone disease; MetS, metabolic syndrome; NAFLD, nonalcoholic fatty liver disease.

FigureS3. GRADE evidence


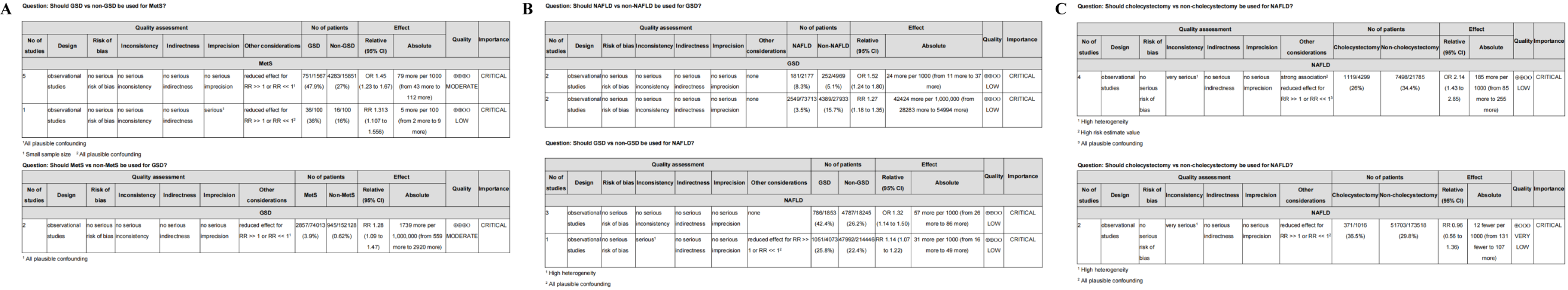


**(A)** The GRADE evidence of “Should GSD vs non-GSD be used for MetS” and “Should MetS vs non-MetS be used for GSD” (including cross-sectional study and prospective cohort study); **(B)** The GRADE evidence of “Should NAFLD vs non-NAFLD be used for GSD” and “Should GSD vs non-GSD be used for NAFLD” (including cross-sectional study and prospective cohort study); **(C)** The GRADE evidence of “Should cholecystectomy vs non-cholecystectomy be used for NAFLD” and “Should cholecystectomy vs non-cholecystectomy be used for NAFLD” (including cross-sectional study and prospective cohort study).

Abbreviations: GSD, gallstone disease; MetS, metabolic syndrome; NAFLD, nonalcoholic fatty liver disease.

Table S1 Search strategy for literatures about the association of gallstone disease with metabolic syndrome and non-alcoholic fatty liver disease and the association of cholecystectomy with non-alcoholic fatty liver disease

| Medline |  | Search strings |  | Results |
| --- | --- | --- | --- | --- |
| 1 |  | cholelithiasis [MeSH Terms] |  | 37163 |
| 2 |  | gallstone disease [Text Word] |  | 2891 |
| 3 |  | 1 OR 2 |  | 38002 |
| 4 |  | metabolic syndrome [MeSH Terms] |  | 36603 |
| 5 |  | syndrome X [Text Word] |  | 2174 |
| 6 |  | insulin resistance syndrome [Text Word] |  | 1719 |
| 7 |  | MetS [Text Word] |  | 13832 |
| 8 |  | 4 OR 5 OR 6 OR 7 |  | 45244 |
| 9 |  | non-alcoholic fatty liver disease [MeSH Terms] |  | 19954 |
| 10 |  | nonalcoholic Steatohepatitis [Text Word] |  | 7132 |
| 11 |  | NAFLD [Text Word] |  | 21259 |
| 12 |  | NASH [Text Word] |  | 11769 |
| 13 |  | 9 OR 10 OR 11 OR 12 |  | 33227 |
| 14 |  | cholecystectomy [MeSH Terms] |  | 30262 |
| 15 |  | Laparoscopic cholecystectomy [MeSH Terms] |  | 13332 |
| 16 |  | 14 OR 15 |  | 30262 |
| 17 |  | 3 AND 8 |  | 56 |
| 18 |  | 3 AND 13 |  | 88 |
| 19 |  | 13 AND 16 |  | 30 |
|  |  |  |  |  |
| Embase |  |  |  |  |
| 1 |  | 'cholelithiasis'/exp OR cholelithiasis |  | 68150 |
| 2 |  | 'gallstone disease' |  | 28451 |
| 3 |  | 1 OR 2 |  | 70398 |
| 4 |  | 'metabolic syndrome X'/exp OR 'metabolic syndrome X' |  | 94056 |
| 5 |  | 'syndrome x' |  | 330971 |
| 6 |  | 'insulin resistance syndrome' |  | 50266 |
| 7 |  | 'MetS' |  | 23774 |
| 8 |  | 4 OR 5 OR 6 OR 7 |  | 359523 |
| 9 |  | 'nonalcoholic fatty liver'/exp OR 'nonalcoholic fatty liver' |  | 58787 |
| 10 |  | 'nonalcoholic AND ('steatohepatitis'/exp OR steatohepatitis) |  | 25744 |
| 11 |  | 'NAFLD' |  | 36964 |
| 12 |  | 'NASH' |  | 36603 |
| 13 |  | 9 OR 10 OR 11 OR 12 |  | 83002 |
| 14 |  | 'cholecystectomy'/exp OR cholecystectomy |  | 65860 |
| 15 |  | 'laparoscopic cholecystectomy'/exp OR 'laparoscopic cholecystectomy' |  | 7859 |
| 16 |  | 14 OR 15 |  | 65860 |
| 17 |  | 3 AND 8 |  | 1139 |
| 18 |  | 3 AND 13 |  | 583 |
| 19 |  | 13 AND 16 |  | 286 |
|  |  |  |  |  |
| Web of Science |  |  |  |  |
| 1 |  | Topic=('cholelithiasis') |  | 35999 |
| 2 |  | Topic= ('gallstone disease') |  | 19191 |
| 3 |  | 1 OR 2 |  | 46076 |
| 4 |  | Topic= ('metabolic syndrome') |  | 330345 |
| 5 |  | Topic= ('syndrome X') |  | 149073 |
| 6 |  | Topic= ('insulin resistance syndrome') |  | 78300 |
| 7 |  | Topic= ('MetS') |  | 16851 |
| 8 |  | 4 OR 5 OR 6 OR 7 |  | 468268 |
| 9 |  | Topic= ('nonalcoholic fatty liver') |  | 31866 |
| 10 |  | Topic= ('nonalcoholic Steatohepatitis') |  | 20829 |
| 11 |  | Topic= ('NAFLD') |  | 35539 |
| 12 |  | Topic= ('NASH') |  | 53895 |
| 13 |  | 9 OR 10 OR 11 OR 12 |  | 93296 |
| 14 |  | Topic= ('cholecystectomy') |  | 58483 |
| 15 |  | Topic= ('laparoscopic cholecystectomy') |  | 30494 |
| 16 |  | 14 OR 15 |  | 58483 |
| 17 |  | 3 AND 8 |  | 1434 |
| 18 |  | 3 AND 13 |  | 341 |
| 19 |  | 13 AND 16 |  | 146 |

Table S2 Check List for Quality Assessment and Scoring of Nonrandomized Studies

| Check List |
| --- |
| Selection |
| 1. How representative was the selected group in comparison with the general community population? (if yes, one star; no star if the participants were selected or  selection of group was not described) |
| 2. How representative was the group with GSD/MetS/ NAFLD/cholecystectomy in comparison with the group without GSD/MetS/ cholecystectomy? (if drawn from the same community, one star; no star if drawn from a different source or selection of group was not described) |
| 3. Ascertainment of high-risk group in exposure of GSD/MetS/NAFLD/cholecystectomy (if yes, one star) |
| 4. Demonstration that the disease (GSD/MetS/NAFLD) outcome was not present at start of study (if yes, one star) |
| Comparability |
| 5. Comparison was controlled for age and gender (if yes, one star; no star was assigned if the two groups differed) |
| 6. Comparison was controlled for 2 or more variables including alcohol intake, cigarette smoking, family history (one star was assigned as if two or more of these three characteristics were controlled for; no star was assigned if one or less characteristic was controlled for) |
| Outcome assessment |
| 7. Clearly defined disease (GSD/MetS/NAFLD) outcome by certain criteria (yes, one star for information ascertained in literature; no star if this information was not  reported) |
| 8. Adequate evaluation of observation of ensuing disease (GSD/MetS/NAFLD) outcome (if yes, one star) |
| 9. Adequacy of follow-up of objects (one star if follow-up rate > 90%) |

Abbreviations: GSD, gallstone disease; MetS, metabolic syndrome; NAFLD, nonalcoholic fatty liver disease.

Table S3 Quality assessment of the studies included in the meta-analysis by NOS*

| NOS scale | Kim et al. 2021 (1) | Chen et al. 2012 (2) | Nahum et al. 2005 (3) | Zhu et al. 2016 (4) | Lin et al.2014 (5) | Naim et al. 2011 (6) | Amit et al. 2019 (7) | Kim et al.  2009 (8) |
| --- | --- | --- | --- | --- | --- | --- | --- | --- |
| **A Selection (maximum 4)**  **1.Representativeness of general community population**  **2.Subjects with GSD/MetS were drawn from the same community**  **3.Ascertainment the exposure of GSD/MetS**  **4.MetS/GSD was not present at baseline**  **B Comparability (maximum 2)**  **5.Controlled for age and gender**  **6.Controlled for 2 or more variables including alcohol intake, cigarette smoking, family history**  **C Outcome (maximum 3)**  **7. Clearly defined MetS/GSD outcome by certain criteria**  **8.** **Adequacy of the evaluation of the results**  **9.** **Adequacy of follow-up rate (>90%) of objects**  **Total scores (maximum 9)** | 1  1  1  1  0  1  1  1  1  8 | 1  1  1  0  1  0  1  1  1  7 | 1  1  1  0  1  0  1  1  1  7 | 1  1  1  1  1  1  1  1  1  9 | 1  1  1  0  1  0  1  1  1  7 | 1  1  1  0  0  0  1  1  1  6 | 1  1  1  1  1  0  1  1  1  8 | 1  1  1  1  0  0  1  1  1  7 |

Continued

| NOS scale | Koller et al. 2012 (9) | LEE et al.  2014 (10) | Chang et al. 2018(11) | Kim et al.  2019 (12) | Liu et al.  2014 (13) | Qiao et al.  2009 (14) |
| --- | --- | --- | --- | --- | --- | --- |
| **A Selection (maximum 4)**  **1.Representativeness of general community population**  **2.Subjects with GSD/NAFLD were drawn from the same community**  **3.Ascertainment the exposure of** **GSD/NAFLD**  **4. GSD/NAFLD was not present at baseline**  **B Comparability (maximum 2)**  **5.Controlled for age and gender**  **6.Controlled for 2 or more variables including alcohol intake, cigarette smoking, family history**  **C Outcome (maximum 3)**  **7. Clearly defined GSD/NAFLD outcome by certain criteria**  **8. Adequacy of the evaluation of the results**  **9. Adequacy of follow-up rate (>90%) of objects**    **Total scores (maximum 9)** | 1  1  1  0  0  1  1  1  1  7 | 1  1  1  1  0  0  1  1  1  7 | 1  1  1  1  0  1  1  1  1  8 | 1  1  1  1  0  0  1  1  1  7 | 1  1  1  1  0  1  1  1  1  8 | 1  1  1  0  0  0  1  1  1  6 |

Continued

| NOS scale | Kwak  al.2015 (15) | Chang  et al. 2018(11) | Yun et al.  2016 (16) | Carmen  et al. 2020 (17) | Yue  et al.2019 (18) | Ruh et al.  2012 (19) |  |
| --- | --- | --- | --- | --- | --- | --- | --- |
| **A Selection (maximum 4)**  **1.Representativeness of general community population**  **2.Subjects with cholecystectomy were drawn from the same community**  **3.Ascertainment the exposure of cholecystectomy**  **4.NAFLD was not present at baseline**  **B Comparability (maximum 2)**  **5.Controlled for age and gender**  **6.Controlled for 2 or more variables including alcohol intake, cigarette smoking, family history**  **C Outcome (maximum 3)**  **7. Clearly defined GSD outcome by certain criteria**  **8. Adequacy of the evaluation of the results**  **9. Adequacy of follow-up rate (>90%) of objects**    **Total scores (maximum 9)** | 1  1  1  1  1  1  1  1  1  9 | 1  1  1  1  0  1  1  1  1  8 | 1  1  1  0  0  0  1  1  1  6 | 1  1  1  1  1  1  1  1  1  9 | 1  1  1  0  1  1  1  1  1  8 | 1  1  1  0  1  0  1  1  1  7 |  |

* NOS: Newcastle-Ottawa Scale

“1” meant the study was corresponded to the NOS criteria, “0” meant the study wasn’t corresponded to the NOS criteria

Abbreviations: GSD, gallstone disease; MetS, metabolic syndrome; NAFLD, nonalcoholic fatty liver disease.

Table S4 Definition of MetS and its Related Components in Enrolled Studies

| Author,  publication(ref) | Obesity | Hypertriglyceridemia | low HDL-C | Hyperglycemia | Hypertension | Diagnostic criteria | MetS definition |
| --- | --- | --- | --- | --- | --- | --- | --- |
| Kim et al.  2021 (1) | WC≥90cm for men  WC≥85cm for women | TG≥1.695 mmol/L | HDL-C＜1.036mmol/L  for men  HDL-C＜1.295mmol/L  for women | FBG≥5.55 mmol/L | SBP≥130mmHg  and/or  DBP≥85mmHg | three of the following five criteria were grounds for definition | IDF criteria  (21) |
| Chen et al.  2012 (2) | WC≥90cm for men  WC≥80cm for women | TG≥150mg/dL  (1.7 mmol/L)  or medical treatment for elevated TG | HDL-C＜40  mg/dL (1.03mmol/L)  for men  HDL-C＜50  mg/dL (1.29mmol/L)  for women  or medication for low HDL-C | FBG≥100mg/dL  (5.6 mmol/L)  or  medication for elevated blood glucose | SBP≥130mmHg  and/or  DBP≥85mmHg  or medication for high blood pressure | three of the following five criteria were grounds for definition | NCEP-ATP-III on the Asia criteria  (22) |
| Nahum et al.  2005 (3) | WC＞102cm for men  WC＞88cm for women | TG≥1.7 mmol/L | HDL-C＜1.03mmol/L for men  HDL-C＜1.3mmol/L for women | FBG≥6.1mmol/L | SBP≥17.3kPa  and/or  DBP≥11.4kPa | three of the following five criteria were grounds for definition | NCEP-ATPIII criteria  (23) |
| Zhu et al.  2016 (4) | BMI≥25.0 kg/m^2^ | TG≥1.7 mmol/L  (110 mg/dL) | fasting HDL＜0.9 mmol/L (35 mg/dL) | FBG≥6.1 mmol/L (110 mg/dl) or  2 h PG≥7.8 mmol/L (140mg/dl) or previous diagnosis | SBP≥140 mm Hg  and/or  DBP≥90mm Hg  or previous diagnosis | three of the following five criteria were grounds for definition | CDS criteria  (24) |
| Lin et al.  2014 (5) | WC≥90cm for men  WC≥80cm for women | TG≥150mg/dL | HDL-C<40mg/dL for men  HDL-C＜50mg/dL for women | FBG≥100 mg/dL  or DM  history (including self-reported or medical record) | SBP≥130mm Hg  or DBP≥85mm Hg  or hypertension history  (including self-reported or  medical record） | three of the following five criteria were grounds for definition | Taiwan National Health Department criteria  (25) |
| Naim et al.  2011 (6) | WC≥102cm for men  WC≥88cm for women | TG≥1.695 mmol/L | HDL-C＜1.036mmol/L for men  HDL-C＜1.295mmol/L for women | FBG≥6.105 mmol/L | SBP≥130mm Hg  and DBP≥85mm Hg | three of the following five criteria were grounds for definition | NCEP-ATPIII criteria  (23) |
| Amit et al.  2019 (7) | WC≥102cm for men  WC≥88cm for women | TG≥150mg/dL | HDL-C＜40 mg/dL for men  HDL-C＜50mg/dL for women | FBG≥110mg/dL | SBP≥130mm Hg  And/or  DBP≥85mm Hg | three of the following five criteria were grounds for definition | NCEP-ATPIII criteria  (23) |
| Kim et al.  2009 (8) | BMI≥25.0 kg/m^2^ | TG≥150mg/dL | HDL-C＜40 mg/dL for men  HDL-C＜50mg/dL for women | FBG≥110mg/dL | SBP≥130mm Hg  And/or  DBP≥85mm Hg | three of the following five criteria were grounds for definition | NCEP-ATPIII criteria  (23) |

Abbreviations: WC, waist circumference; BMI, body mass index; TG, triglyceride；HDL-C, high density lipoprotein cholesterol; FBG, fasting blood glucose; PG: post-meal glucose; DM, diabetes mellitus; SBP, systolic blood pressure; DBP, diastolic blood pressure; IDF, International Diabetes Federation; NCEP ATPIII, National Cholesterol Education Program Adult Treatment Group III; CDS, Chinese Diabetes Society.

**References**

1. Kim Y, Oh CM, Ha E, Park SK, Jung JY, Ryoo JH. Association between metabolic syndrome and incidence of cholelithiasis in the Korean population. J Gastroenterol Hepatol. (2021);36(12):3524-31. doi:10.1111/jgh.15568

2. Chen LY, Qiao QH, Zhang SC, Chen YH, Chao GQ, Fang LZ. Metabolic syndrome and gallstone disease. World J Gastroenterol. (2012);18(31):4215-20. doi:10.3748/wjg.v18.i31.4215

3. Méndez-Sánchez N, Chavez-Tapia NC, Motola-Kuba D, Sanchez-Lara K, Ponciano-Rodríguez G, Baptista H, et al. Metabolic syndrome as a risk factor for gallstone disease. World J Gastroenterol. (2005);11(11):1653-7. doi:10.3748/wjg.v11.i11.1653

4. Zhu Q, Sun X, Ji X, Zhu L, Xu J, Wang C, et al. The association between gallstones and metabolic syndrome in urban Han Chinese: a longitudinal cohort study. Sci Rep. (2016);6:29937. doi:10.1038/srep29937

5. Lin IC, Yang YW, Wu MF, Yeh YH, Liou JC, Lin YL, et al. The association of metabolic syndrome and its factors with gallstone disease. BMC Fam Pract. (2014);15:138. doi:10.1186/1471-2296-15-138

6. Ata N, Kucukazman M, Yavuz B, Bulus H, Dal K, Ertugrul DT, et al. The metabolic syndrome is associated with complicated gallstone disease. Can J Gastroenterol. (2011);25(5):274-6. doi:10.1155/2011/356761

7. Peswani AR, Sequeira VJ, D’silva M, Ghanwat S, Shah PP, Pinto AC. Association between gallstone disease and metabolic syndrome. IJCMR. (2019);6(10):J1-J5. doi:10.21276/ijcmr.2019.6.10.13

8. Kim J, Lee K. Relationship between Metabolic Syndrome and Gallbladder Stone. Korean Journal of Family Medicine. (2009);30(8):610-6. doi:doi.org/10.4082/kjfm.2009.30.8.610

9. Koller T, Kollerova J, Hlavaty T, Huorka M, Payer J. Cholelithiasis and markers of nonalcoholic fatty liver disease in patients with metabolic risk factors. Scand J Gastroenterol. (2012);47(2):197-203. doi:10.3109/00365521.2011.643481

10. Lee YC, Wu JS, Yang YC, Chang CS, Lu FH, Chang CJ. Moderate to severe, but not mild, nonalcoholic fatty liver disease associated with increased risk of gallstone disease. Scand J Gastroenterol. (2014);49(8):1001-6. doi:10.3109/00365521.2014.920912

11. Chang Y, Noh YH, Suh BS, Kim Y, Sung E, Jung HS, et al. Bidirectional Association between Nonalcoholic Fatty Liver Disease and Gallstone Disease: A Cohort Study. J Clin Med. (2018);7(11). doi:10.3390/jcm7110458

12. Kim YK, Kwon OS, Her KH. The grade of nonalcoholic fatty liver disease is an independent risk factor for gallstone disease: An observational Study. Medicine (Baltimore). (2019);98(27):e16018. doi:10.1097/md.0000000000016018

13. Liu J, Lin H, Zhang C, Wang L, Wu S, Zhang D, et al. Non-alcoholic fatty liver disease associated with gallstones in females rather than males: a longitudinal cohort study in Chinese urban population. BMC Gastroenterol. (2014);14:213. doi:10.1186/s12876-014-0213-y

14. Qiao QH, Zhu WH, Yu YX, Huang FF, Chen LY. Nonalcoholic fatty liver was associated with asymptomatic gallstones in a Chinese population. Medicine (Baltimore). (2017);96(38):e7853. doi:10.1097/md.0000000000007853

15. Kwak M-S, Kim D, Chung GE, Kim W, Kim YJ, Yoon J-H. Cholecystectomy is independently associated with nonalcoholic fatty liver disease in an Asian population. World Journal of Gastroenterology: WJG. (2015);21(20):6287. doi:10.3748/wjg.v21.i20.6287

16. Yun S, Choi D, Lee KG, Kim HJ, Kang BK, Kim H, et al. Cholecystectomy Causes Ultrasound Evidence of Increased Hepatic Steatosis. World J Surg. (2016);40(6):1412-21. doi:10.1007/s00268-015-3396-7

17. Latenstein CSS, Alferink LJM, Darwish Murad S, Drenth JPH, van Laarhoven C, de Reuver PR. The Association Between Cholecystectomy, Metabolic Syndrome, and Nonalcoholic Fatty Liver Disease: A Population-Based Study. Clin Transl Gastroenterol. (2020);11(4):e00170. doi:10.14309/ctg.0000000000000170

18. Yue W, Sun X, Du T. Cholecystectomy versus central obesity or insulin resistance in relation to the risk of nonalcoholic fatty liver disease: the third US National Health and Nutrition Examination Survey. BMC Endocr Disord. (2019);19(1):95. doi:10.1186/s12902-019-0423-y

19. Ruhl CE, Everhart JE. Relationship of non-alcoholic fatty liver disease with cholecystectomy in the US population. Am J Gastroenterol. (2013);108(6):952-8. doi:10.1038/ajg.2013.70

20. Hajong R, Dhal MR, Naku N, Kapa B. Incidence of nonalcoholic fatty liver disease in patients undergoing laparoscopic cholecystectomy. J Family Med Prim Care. (2018);7(6):1375-8. doi:10.4103/jfmpc.jfmpc_193_18

21. Alberti KG, Eckel RH, Grundy SM, Zimmet PZ, Cleeman JI, Donato KA, et al. Harmonizing the metabolic syndrome: a joint interim statement of the International Diabetes Federation Task Force on Epidemiology and Prevention; National Heart, Lung, and Blood Institute; American Heart Association; World Heart Federation; International Atherosclerosis Society; and International Association for the Study of Obesity. Circulation. (2009);120(16):1640-5. doi:10.1161/circulationaha.109.192644

22. Heng D, Ma S, Lee JJ, Tai BC, Mak KH, Hughes K, et al. Modification of the NCEP ATP III definitions of the metabolic syndrome for use in Asians identifies individuals at risk of ischemic heart disease. Atherosclerosis. (2006);186(2):367-73. doi:10.1016/j.atherosclerosis.2005.07.020

23. Executive Summary of The Third Report of The National Cholesterol Education Program (NCEP) Expert Panel on Detection, Evaluation, And Treatment of High Blood Cholesterol In Adults (Adult Treatment Panel III). Jama. (2001);285(19):2486-97. doi:10.1001/jama.285.19.2486

24. Lu Y, Lu J, Wang S, Li C, Liu L, Zheng R, et al. Comparison of the diagnostic criteria of metabolic syndrome by International Diabetes Federation and that by Chinese Medical Association Diabetes Branch. Zhonghua yi xue za zhi. (2006);86(6):386-9.

25. Tsai T-Y, Cheng J-F, Lai Y-M. Prevalence of metabolic syndrome and related factors in Taiwanese high-tech industry workers. Clinics. (2011);66:1531-5. doi:10.1590/S1807-59322011000900004
